# SPIKING A Sense of Belonging: Utilizing a Communication Model to Unlock Your Story With Authenticity

**DOI:** 10.15766/mep_2374-8265.11567

**Published:** 2025-12-30

**Authors:** Natalie Jane Koons, Ernest Okwuonu, Rameshwar R. Rao, Ruth Belay, Al'ai Alvarez, Margarita Ramos, Natalie Coupe, Lahia Yemane

**Affiliations:** 1 Anesthesiology Resident, Department of Anesthesiology, Perioperative, and Pain Medicine, Stanford University School of Medicine; 2 Attending General and Consultation-Liaison Psychiatrist, Comprehensive Psychiatric Services, Stanford Health Care–Tri-Valley and Sutter Health; 3 Acting Assistant Professor, Ben Towne Center for Childhood Cancer and Blood Disorders Research, Seattle Children's Research Institute; Acting Assistant Professor, Division of Pediatric Hematology, Oncology, Bone Marrow Transplant, and Cellular Therapies, Department of Pediatrics, University of Washington School of Medicine; 4 Clinical Assistant Professor, Department of Urology, Stanford University School of Medicine; 5 Clinical Associate Professor and Director of Well-Being, Department of Emergency Medicine, Stanford University School of Medicine; 6 Assistant Professor, Division of Hospital Medicine, Children's National Hospital and George Washington University School of Medicine and Health Sciences; 7 Critical Care Medicine Division Coordinator, Department of Anesthesiology, Perioperative and Pain Medicine, Stanford University School of Medicine; 8 Clinical Professor, Division of General Pediatrics, Department of Pediatrics, Stanford University School of Medicine

**Keywords:** Belonging, Uniqueness, Authenticity, Storytelling, Workplace Culture, Communication Skills, Law & Medicine

## Abstract

**Introduction:**

Balancing belonging with uniqueness, particularly in diverse group settings like medicine, remains a challenge that can threaten inclusion. Storytelling can be a powerful way to cultivate inclusion. However, many individuals lack frameworks, language, or confidence to share their stories in ways that feel both authentic and professionally appropriate in the workplace. We addressed this gap by teaching participants how to apply an adaptation to the evidence-based SPIKES (Setting, Perception, Invitation, Knowledge, Emotions, and Strategize or Summarize) model of communication to their own stories to facilitate an increased sense of uniqueness and belonging.

**Methods:**

We developed an interactive 60-minute workshop geared toward learners, faculty, and educational administrators that included didactics, reflection exercises, and storytelling using an adapted SPIKES model of communication. To assess the workshop's impact, we administered a postworkshop evaluation. We analyzed Likert-scale questions using descriptive statistics and conducted content analysis of open-ended prompts.

**Results:**

The workshop was presented three times at Stanford Medicine in-person conferences. Of the 75 participants, 64 completed a postworkshop survey, resulting in an 85% response rate. Overall, 94% of respondents *agreed* or *strongly agreed* that the workshop achieved its educational objectives, and 92% felt it was a valuable use of their time. Key themes in participants’ intended behavior changes included sharing personal stories to foster a sense of belonging, embracing vulnerability by connecting with others, and using the SPIKES model of communication in everyday conversations.

**Discussion:**

This workshop was effective in applying an adapted SPIKES model of communication to authentic storytelling to cultivate belonging.

## Educational Objectives

By the end of this activity, learners will be able to:

1. Differentiate belonging and uniqueness.
2. Illustrate how sharing personal stories can enhance a sense of belonging in the workplace.
3. Describe how the SPIKES (Setting, Perception, Invitation, Knowledge, Emotions, and Strategize or Summarize) model of communication can be adapted to sharing personal stories.
4. Apply the SPIKES model of communication to share personal stories to foster a sense of authenticity and belonging in the workplace.

## Introduction

Balancing belonging with uniqueness, particularly in diverse group settings like medicine, remains a challenge that can threaten inclusion. Belonging is a fundamental human need and is defined as a subjective feeling that one is an integral part of one's surrounding systems, including the workplace.^1–5^ Belonging can be aligned with one's cultural and subcultural identities and can represent a construct of physical safety and well-being.^2^ There is a distinction between belonging as a core psychological need and a situation-specific sense of belonging that can often lead to being perceived as a need or want to change oneself to assimilate to the culture. On the other hand, uniqueness, defined by Snyder and Fromkin, is the distinctive and differentiated sense of self. In contrast to belonging, uniqueness focuses on the individual rather than establishing and maintaining interpersonal relationships and/or the need to be integrated into a group.^6,7^

Shore and colleagues described a framework of inclusion that proposes that the intersection of uniqueness and belonging creates a feeling of inclusion. Individuals who have a high sense of belonging (i.e., belongingness) and high value in uniqueness are treated as an insider and benefit from being a unique member of a group. On the other end of the framework, those who have a low sense of belongingness and low value in uniqueness are excluded. They are often treated as an outsider, one who is not valued for their unique qualities. Those who have a high sense of belongingness but low value in uniqueness assimilate and conform to organizational or dominant culture norms, which also continue to downplay uniqueness. Those who have a low sense of belongingness but high value in uniqueness are often valued for their unique qualities but are not treated as an insider.^5^ Ely and Thomas’ qualitative study illustrated that work groups who adopted this perspective acknowledged the unique qualities of individuals, but minority members were not considered to be a part of the larger organization and, therefore, were subject to isolation and stereotypes.^8^ Studies show that fostering belonging and uniqueness together benefits diverse team members through increased career optimism,^9^ group collaboration, and the augmentation of individual skills.^8^

In medicine, developing skills to facilitate discussions on topics traditionally thought to be undiscussable that arise in cross-cultural relationships is crucial.^10^ Dr. Adrianne Haggins discusses the critical need to create spaces for collective dialogue among colleagues, medical residents, and students about the intersection of their personal (e.g., race, ethnicity, and gender), and professional identities.^10^ This is crucial to enhancing belonging among women, older physicians, and underrepresented individuals in medicine.^10,11^ While evidence in the literature acknowledges the importance of cross-cultural discussions, it often lacks guidance on leveraging belonging and uniqueness for improved inclusion.

One way to leverage both belonging and uniqueness is by storytelling, or sharing one's personal stories, as this has historically served as a tool for self-reflection, healing, and connecting with others from different cultures.^12^ These stories can forge connections with others, fostering a sense of belonging and understanding of another person's unique perspective and culture. Through storytelling, we invite others to empathize with the challenges, triumphs, and shared personal experiences one may encounter. Yet, while storytelling holds tremendous potential to foster connection and belonging, effectively sharing one's story—particularly in professional, cross-cultural spaces—requires more than willingness alone. Many individuals lack frameworks, language, or confidence to articulate their lived experiences in ways that feel both authentic and professionally appropriate. Just as we train clinicians in communication and procedural skills, we must also intentionally cultivate the skills, provide the tools, and offer structured practice environments that empower individuals to share their narratives with clarity, purpose, and impact.

The SPIKES model of communication (Setting, Perception, Invitation, Knowledge, Emotions, and Strategize or Summarize) was developed initially by Baile and colleagues in 2000 and is a communication tool designed to help health care providers systematically deliver difficult news to patients.^13–15^ Studies have shown that the SPIKES model of communication is associated with improved knowledge and performance, increased confidence, improved communication, and patient care.^14–16^ Recognizing that many individuals struggle with how to structure and share emotionally complex or deeply personal narratives, we saw parallels between the challenges of disclosing difficult news to patients and the vulnerability involved in sharing one's own story—prompting us to adapt the SPIKES model of communication as a supportive framework for how people could share their personal and professional stories to foster a sense of belonging.

*MedEdPORTAL* has several examples of workshops focused on improving communication skills^17,18^ and inclusion as it relates to microaggressions.^19–22^ Still, to date, no workshops described in *MedEdPORTAL* focus on utilizing a model to enhance belonging and uniqueness through sharing personal and professional stories. Our novel workshop addresses this gap by teaching participants how to apply the SPIKES model of communication to their own personal or professional stories to highlight their uniqueness and facilitate an increased sense of authenticity and belonging.

## Methods

### Facilitators

This workshop was developed by a diverse group of medical residents, fellows, faculty, and educational administrators, from different clinical specialties, who participated in the Stanford Medicine Leadership Education in Advancing Diversity (LEAD) Program from 2023 to 2024. These specialties included anesthesiology, urology, psychiatry, pediatrics, internal medicine, and emergency medicine. At a minimum, we had three facilitators available at each workshop presentation, and the variability was dependent on the size of the workshop audience and facilitator availability. The facilitators assigned presenter roles before each presentation, including presenting workshop sections, sharing personal experiences, and facilitating group discussions. All facilitators played a crucial role in workshop creation and could lead any part of the workshop as needed. Facilitators had to be skilled at leading small- and large-group discussions with learners from a variety of backgrounds. However, no specialized training was required to facilitate the workshop aside from a review of the materials to familiarize oneself with the specific content beforehand.

### Target Audience

The workshop's target audience included learners of all levels (premedical undergraduate students, medical students, residents, fellows), faculty, and educational administrators.

### Workshop

The workshop was designed using Kern's six-step model for curriculum development.^23^ For the first and second steps, we discussed the challenges of fostering workplace belonging through our collective and diverse experiences as an author group. We then conducted a general needs assessment through a literature review and discussions with other residents, fellows, faculty, and educational administrators in the LEAD Program. For the third step, we developed goals and educational objectives for the workshop based on the literature review and what we felt could be accomplished within an educational workshop. The fourth step involved developing our workshop format, including interactive educational strategies like a PowerPoint didactic presentation, interactive polls, small- and large-group debriefs, and silent reflections. For the fifth step, we presented in-person workshops at various educational conferences with the targeted audience as noted previously. For the final step, we created a postworkshop evaluation to assess the session's effectiveness. The workshop was presented through Stanford Medicine sponsored conferences that were advertised via email, offering participants interested in the topic the option to participate in the workshop. All workshop participants were eligible to complete the postworkshop evaluation, which was optional (i.e., completion of the survey was not required for workshop participation).

We modified and improved the workshop between presentations using feedback from the postworkshop evaluations. We initially developed and presented the workshop as a 75-minute presentation for a forum focused on diversity and inclusion in medicine and then adapted it to a 60-minute presentation to fit the allotted time provided for two additional forums. Major adaptations included shortening each presenter's application of the SPIKES model of communication to their own story to allow more time for small- and large-group discussions and creating an adapted SPIKES model of communication handout for the audience to reference throughout the workshop. Given that the workshop encourages participants to share personal stories, we also asked facilitators to provide examples in the small- or large-group discussions to model vulnerability if participants seemed hesitant to share. We also used a mix of configurations for sharing reflections, such that participants could share with the person sitting next to them or in small- or large-group formats, thereby enabling them to engage in the group size most comfortable to them.

We delivered the workshop using a PowerPoint presentation (Appendix A) and followed the agenda outlined in the facilitator guide (Appendix B), a step-by-step guide handout (Appendix C), and presenter script (Appendix D). The session began with an introduction of the workshop title, authors, and educational objectives. We started the workshop with an interactive group activity where participants shared something about themselves (for example, “I am an anesthesiology resident from Los Angeles, CA.”), first with their peers in small group and then voluntarily with the larger group, by hand-writing their responses on an index card or piece of paper. At the workshop's onset, we also asked participants to reflect on what belonging means to them. Again, they were asked to share with peers and the larger group. We then transitioned to a short didactic portion, first distinguishing between the concepts of belonging and uniqueness and how they complement each other using the framework for inclusion proposed by Shore and colleagues.^5^ We also illustrated how sharing personal experiences through storytelling enhances a sense of belonging and authenticity in the workplace. Following the didactic portion, we prompted participants to reflect on and share a story that was meaningful to them. We asked participants to voluntarily share with the person sitting beside them for small-group and peer-to-peer discussions. Participants were offered the opportunity to share those responses with the larger group if they felt comfortable.

Facilitators then transitioned to explaining the history of the SPIKES model of communication,^14^ the elements of the framework, how it has been utilized, and opportunities for application of the framework to enhance belonging in the workplace. We received written permission from Renato Lenzi, MD, to use and adapt the SPIKES model of communication for this workshop. We then shifted to reviewing how to adapt the SPIKES model of communication to a personal or professional experience and provided participants a step-by-step guide handout (Appendix C) to reference for the rest of the workshop. Next, each facilitator shared a personal story using the SPIKES model of communication, highlighting a meaningful aspect of their experience that aligned with each step of the framework. These reflections illustrated how our individual journeys shape our uniqueness and how shared experiences have deepened our relationships along the way. Facilitators then prompted the audience to again reflect on the meaning of belonging and to consider if their responses have changed after listening to the presenter's application of the SPIKES model of communication with their individual stories.

Participants were then invited to revisit the initial exercises from the beginning of the session and consider how they might reconstruct their narratives using the model of communication, with the goal of fostering vulnerability and authentically sharing aspects of their identity and lived experience. We provided a handout of the SPIKES model of communication for participants to reference while they worked (Appendix C). First, we asked participants to share their stories with two to three others near them and then invited a few to share their stories using the SPIKES model of communication and/or reflections with the large group about the exercise. The workshop concluded with a set of reflection questions for silent contemplation, followed by a call to action inviting the audience to consider how they might apply the SPIKES model of communication in everyday interactions to foster a sense of belonging. We ended with audience questions and time to complete the postworkshop evaluation (Appendix E).

### Evaluation and Analysis

Following each workshop presentation, participants were invited to complete an optional anonymous postworkshop evaluation (Appendix E) either electronically or on paper, depending on the presentation format. The evaluation was adapted for our workshop educational objectives from a template created by medical education leaders in the Stanford Medicine LEAD Program. There was no pilot testing conducted. The survey assessed how well the workshop met its educational objectives using a 5-point Likert scale (1 = *strongly disagree*, 5 = *strongly agree*) and included open-ended questions to gather feedback on strengths and suggestions for improvement. Descriptive statistics were used to analyze quantitative data, and open-ended responses were coded using conventional content analysis by two of the authors (Natalie Koons and Lahia Yemane).

### Institutional Review Board

This study was submitted to the Stanford University Institutional Review Board for review and was determined not to meet the definition of human subject research (protocol number 77687).

## Results

We presented this workshop in-person at three local conferences: (1) Annual Stanford Medicine Diversity & Inclusion Forum, May 2024; (2) Stanford Department of Pathology DEI Mini-Symposium, September 2024; and (3) Annual Stanford University Minority Medical Alliance Conference, February 2025. These were all Stanford Medicine sponsored conferences advertised via email and open to either the Stanford community (conference 2) or broader audiences (conferences 1 and 3). Sixty-four of 75 participants completed a postworkshop evaluation form (85% response rate).

Of the participants who completed the postworkshop evaluation, the majority were medical trainees (48%) and educational administrators (25%), were Asian (27%) or Hispanic (27%), and identified as cisgender women (59%). Full participant demographics are shown in Table 1. Overall, 94% of workshop participants *agreed* or *strongly agreed* that the workshop met its educational objectives, and 92% found it to be a valuable use of their time (Table 2).

**Table 1.**
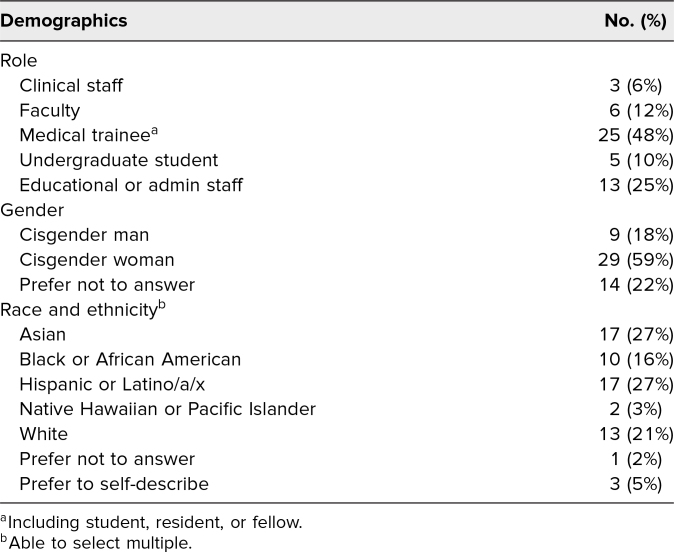
Demographics of the Workshop Participants (*N* = 64) Who Completed the Postworkshop Evaluation

**Table 2.**
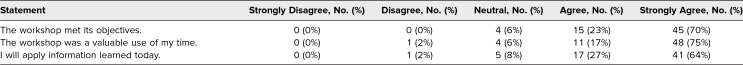
Participant Responses (*N* = 64) to the Postworkshop Evaluation

Qualitative feedback revealed several key themes related to participants’ intended behavior change following the workshop, including sharing personal stories to foster a sense of belonging, embracing vulnerability by engaging with others, and applying the SPIKES model of communication in everyday interactions. Reported barriers to implementation included workplace culture, limited time for reflection, fear or insecurity about sharing personal experiences, and difficulty remembering the SPIKES model of communication. Participants most appreciated the opportunity to hear others’ stories, engage in interactive discussions, and connect the SPIKES model of communication to their own experiences (Table 3).

**Table 3.**
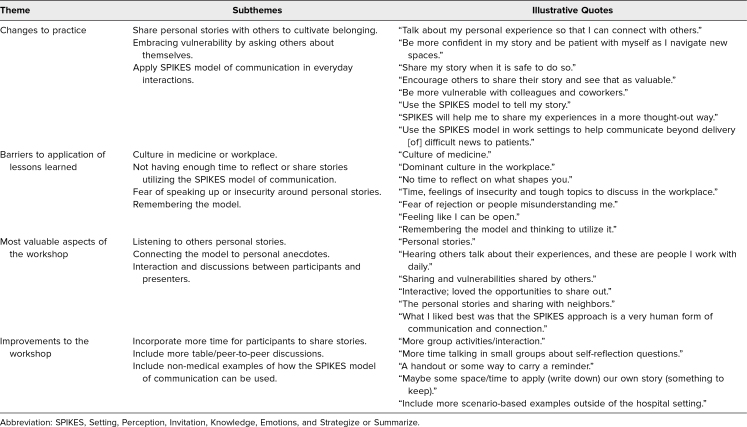
Participant Themes From Open-Ended Questions on Postworkshop Evaluation

## Discussion

We developed and delivered a workshop to teach participants how to apply the SPIKES model of communication to their own personal or professional stories to highlight their uniqueness and facilitate an increased sense of authenticity and belonging. Postworkshop evaluations demonstrated that the majority of participants agreed that each educational objective was met, and that the session was a valuable use of their time. Importantly, participants shared that they planned to apply the SPIKES model of communication in their everyday interactions to foster a sense of belonging and embrace vulnerability in asking others about themselves.

Our workshop addresses a persistent gap in medical education, where there is a lack of inclusive communication strategies taught to medical learners and professions to help them share personal stories. Research shows that underrepresented individuals in academic medicine, especially women and specific racial and ethnic populations, continue to experience disrespect and exclusion.^10^ Existing literature continues to draw attention to the alarming rate of harassment, incivility, and disrespect among underrepresented individuals in academic medicine, and that other racial and ethnic groups are at risk of being targeted.^10^ The crucial need for creating inclusive spaces has been a call-to-action, including developing mechanisms to foster connection to community, specific mentoring programs, and other avenues that could lead to diversifying the curriculum to include activities that foster belonging. In accordance with Shore and colleagues’ framework of inclusion, proposing that the intersection of uniqueness and belonging creates a feeling of inclusion, our workshop fills the gap on leveraging a sense of belonging and uniqueness for improved inclusion in the workplace and within organizational culture.^5^ In response, our approach provides a structured, yet adaptable tool to foster connections and authenticity across disciplines and educational stages.

We presented this workshop for various audiences across our institution, adapting it to accommodate time allotments and based on evaluation feedback. All three sessions were offered to participants in-person only; on two occasions, one of the presenters joined virtually to share their personal story. We think it would be feasible to offer this workshop in a hybrid or fully virtual format as long as there would be online facilitators to lead breakout rooms during the small-group activities. Across all presentations, the most valuable aspects of the workshop were sharing and hearing personal stories and applying the SPIKES model of communication. Therefore, we adjusted the agenda to allow the participants more time to work individually on applying the SPIKES model of communication to their own story and then share with their small group. Also, knowing that this topic can increase feelings of vulnerability, we used a mix of self-reflection, small-group, and large-group activities so that participants could engage to their comfort level.

Although 75 people participated, our postworkshop evaluation response rate was 85%, which may reflect a self-selection bias. Our current evaluation primarily assessed participants’ immediate perceptions of whether the workshop met its educational objectives and perceptions for future behavior change. Future iterations could consider a preworkshop survey that would assess baseline knowledge and use of the SPIKES model of communication in clinical settings, and additional open-ended questions in the postsurvey to explore in what ways a participant felt, if at all, that the adapted SPIKES model of communication helped to better facilitate storytelling. Furthermore, follow-up evaluations could be used to assess retention of knowledge and continued application of the SPIKES model of communication in sharing personal stories, as well as its sustained impact on workplace inclusion. Incorporating long-term implementation and evaluation would provide a more comprehensive understanding of its effectiveness over time. Additionally, our data did not enable us to identify the specific situations, settings, or disciplines for which the workshop may be most effective, although we presented it across a range of settings and audiences. Collecting longitudinal data across diverse environments would help clarify contextual relevance and inform future adaptations of the workshop content.

Storytelling can be a powerful tool to cultivate inclusion within diverse teams in medicine. This workshop was effective in teaching participants how to adapt an established SPIKES model of communication to share one's personal stories to highlight both uniqueness and belonging.

## Appendices


Workshop Presentation.pptxFacilitator Guide.docxHandout.docxPresentation Script.docxEvaluation Form.docx
*All appendices are peer reviewed as integral parts of the Original Publication.*
