# Supplementary material for: SPIKING A Sense of Belonging: Utilizing a Communication Model to Unlock Your Story With Authenticity
Source: MedEdPORTAL. 2025 Dec 30;21:11567. doi: 10.15766/mep_2374-8265.11567 (PMC12748279; doi:10.15766/mep_2374-8265.11567)
Supplement: Supplementary file 1 — Workshop Presentation.pptxFacilitator Guide.docxHandout.docxPresentation Script.docxEvaluation Form.docx [file mep_2374-8265.11567-s001.zip › A. Workshop Presentation.pptx]

## Slide 1
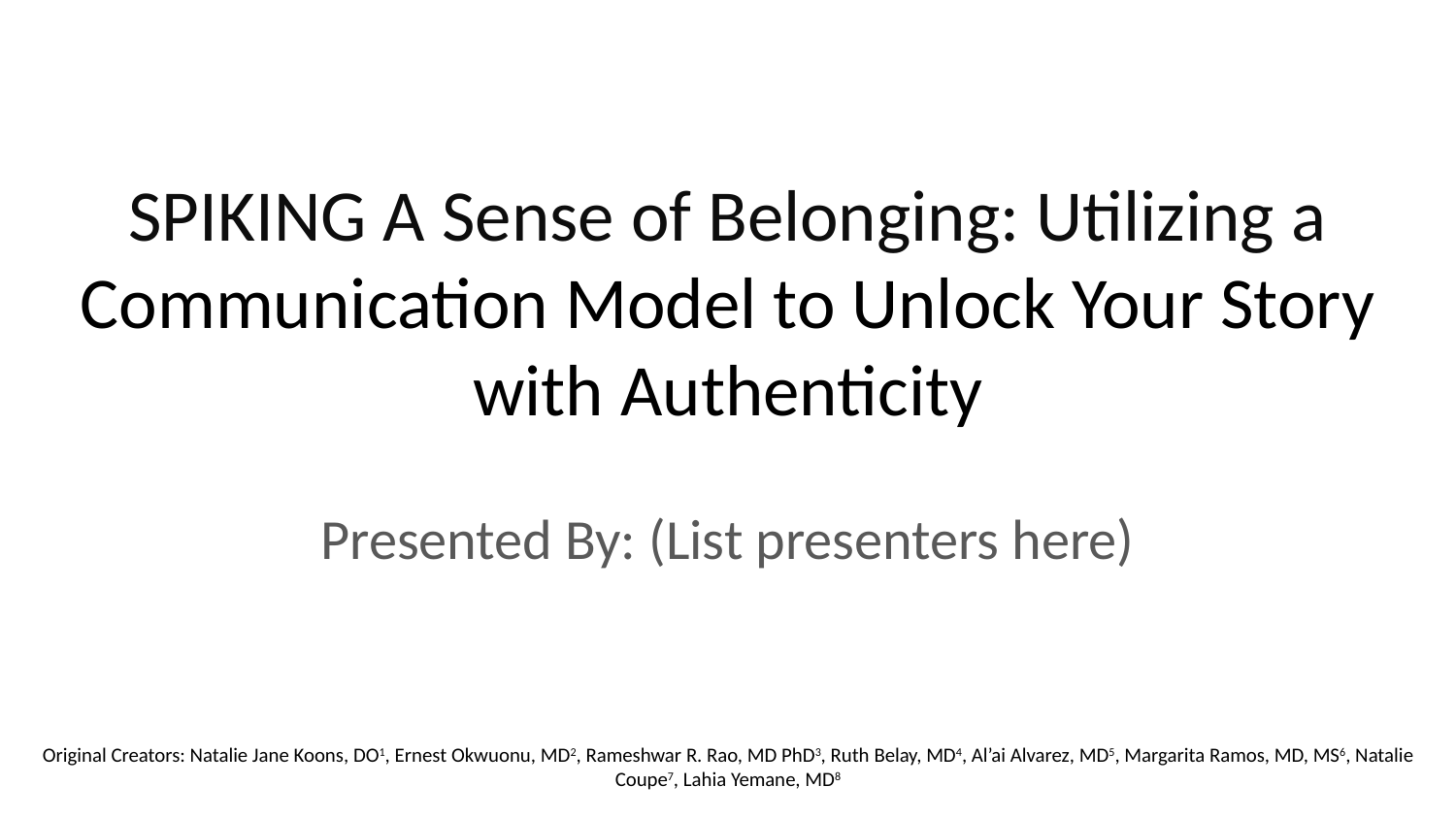

# SPIKING A Sense of Belonging: Utilizing a Communication Model to Unlock Your Story with Authenticity
Presented By: (List presenters here)
Original Creators: Natalie Jane Koons, DO1, Ernest Okwuonu, MD2, Rameshwar R. Rao, MD PhD3, Ruth Belay, MD4, Al’ai Alvarez, MD5, Margarita Ramos, MD, MS6, Natalie Coupe7, Lahia Yemane, MD8

## Slide 2
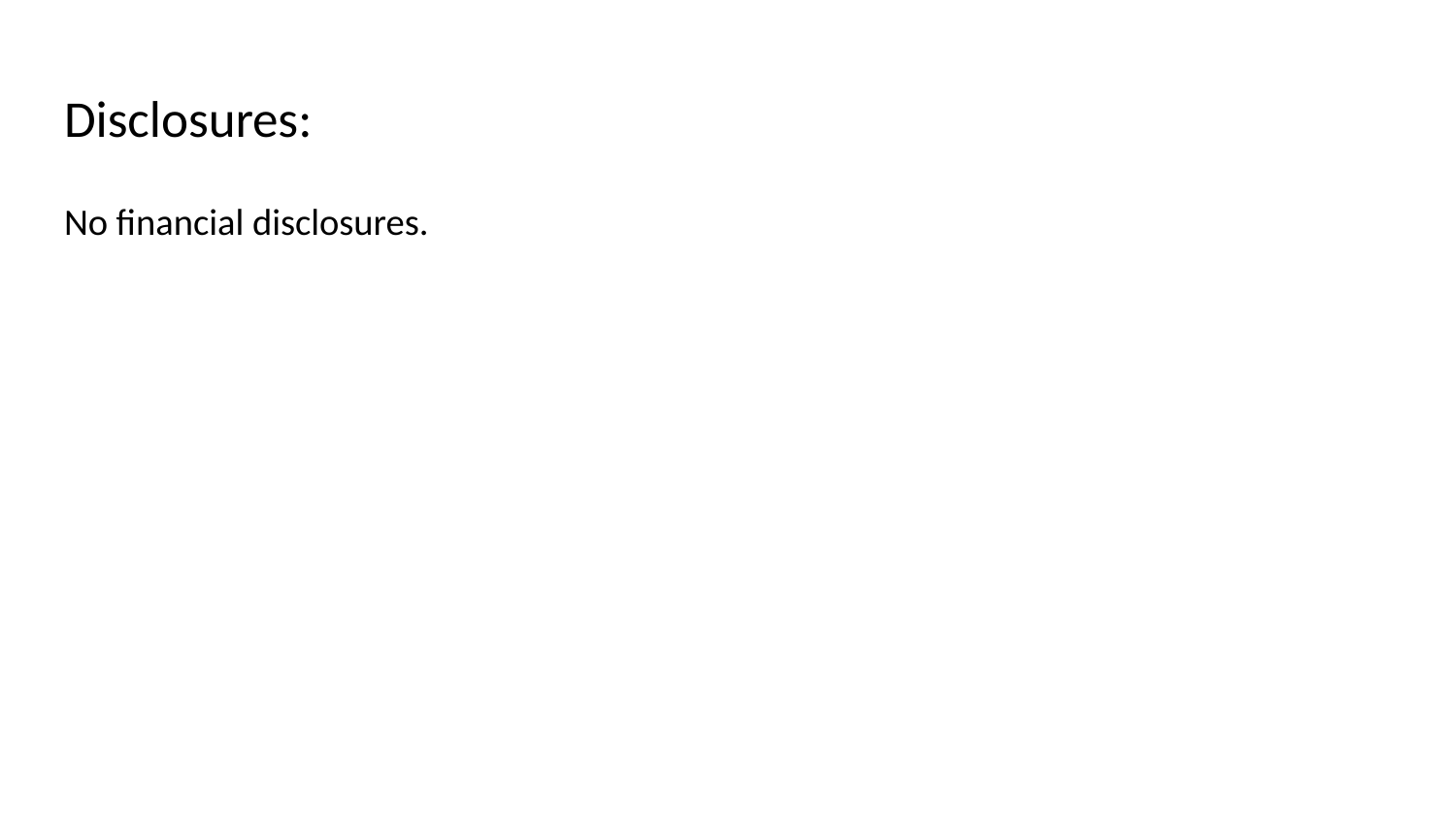

# Disclosures:
No financial disclosures.

## Slide 3
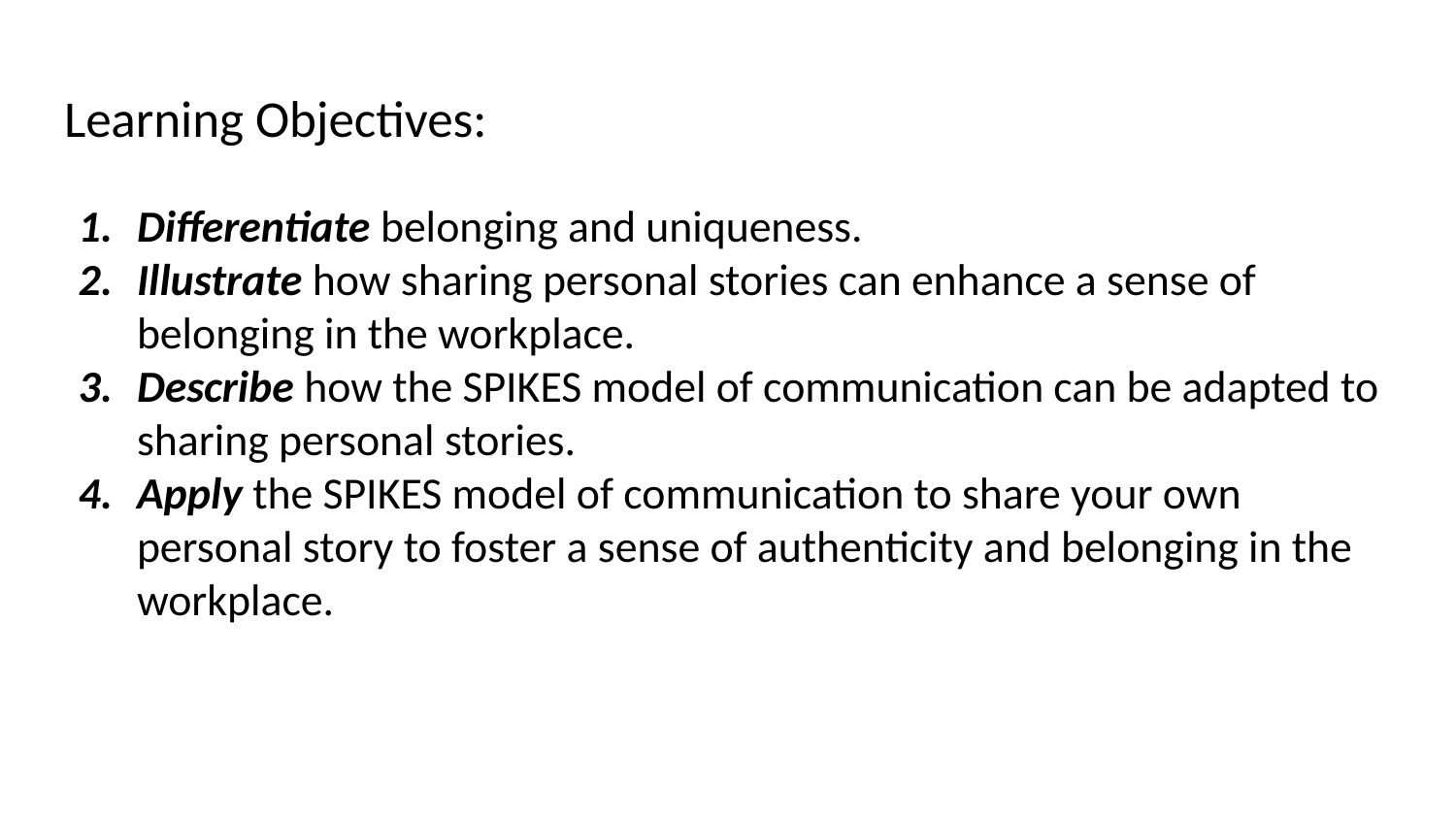

# Learning Objectives:
Differentiate belonging and uniqueness.
Illustrate how sharing personal stories can enhance a sense of belonging in the workplace.
Describe how the SPIKES model of communication can be adapted to sharing personal stories.
Apply the SPIKES model of communication to share your own personal story to foster a sense of authenticity and belonging in the workplace.

## Slide 4
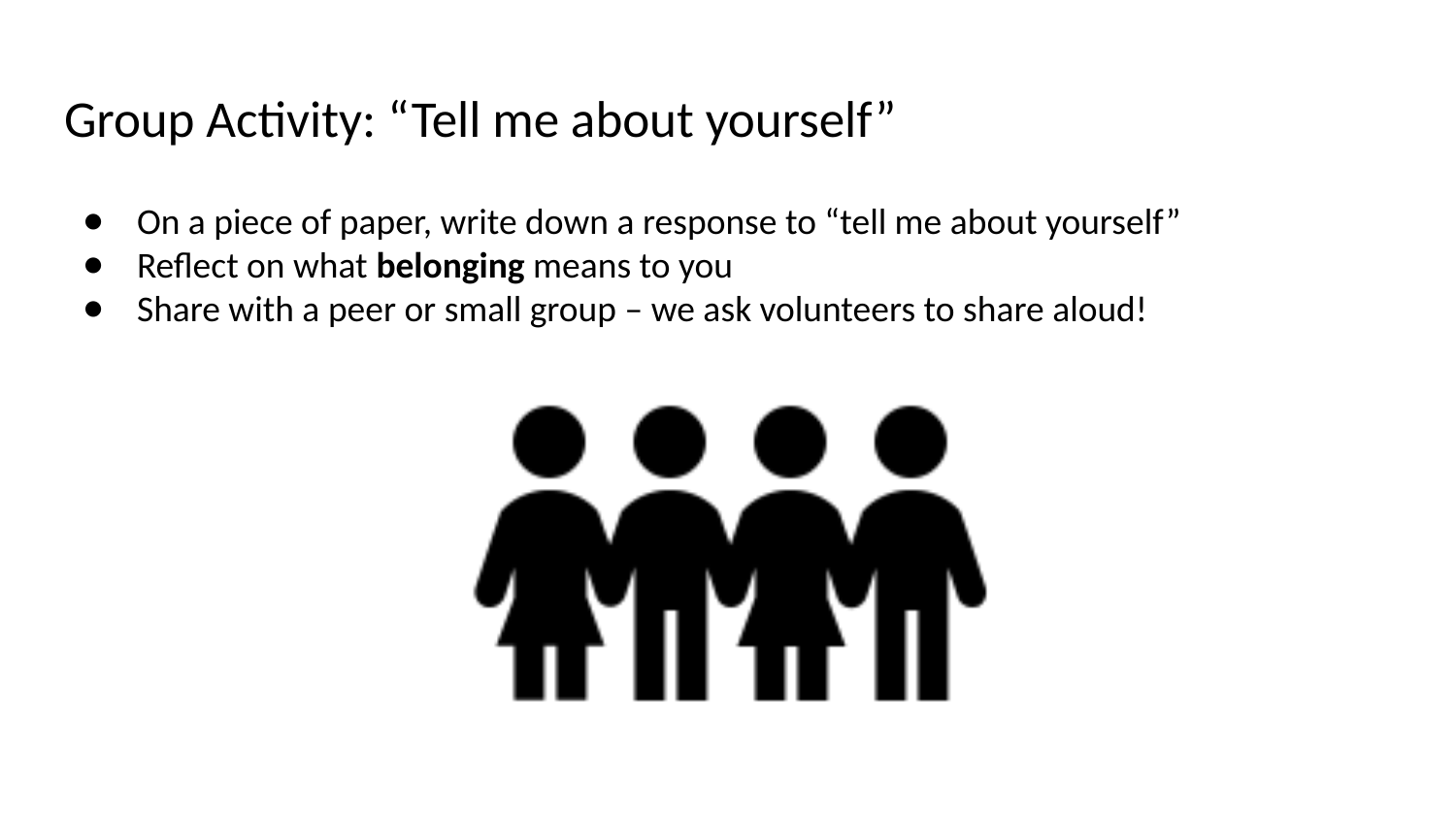

# Group Activity: “Tell me about yourself”
On a piece of paper, write down a response to “tell me about yourself”
Reflect on what belonging means to you
Share with a peer or small group – we ask volunteers to share aloud!

## Slide 5
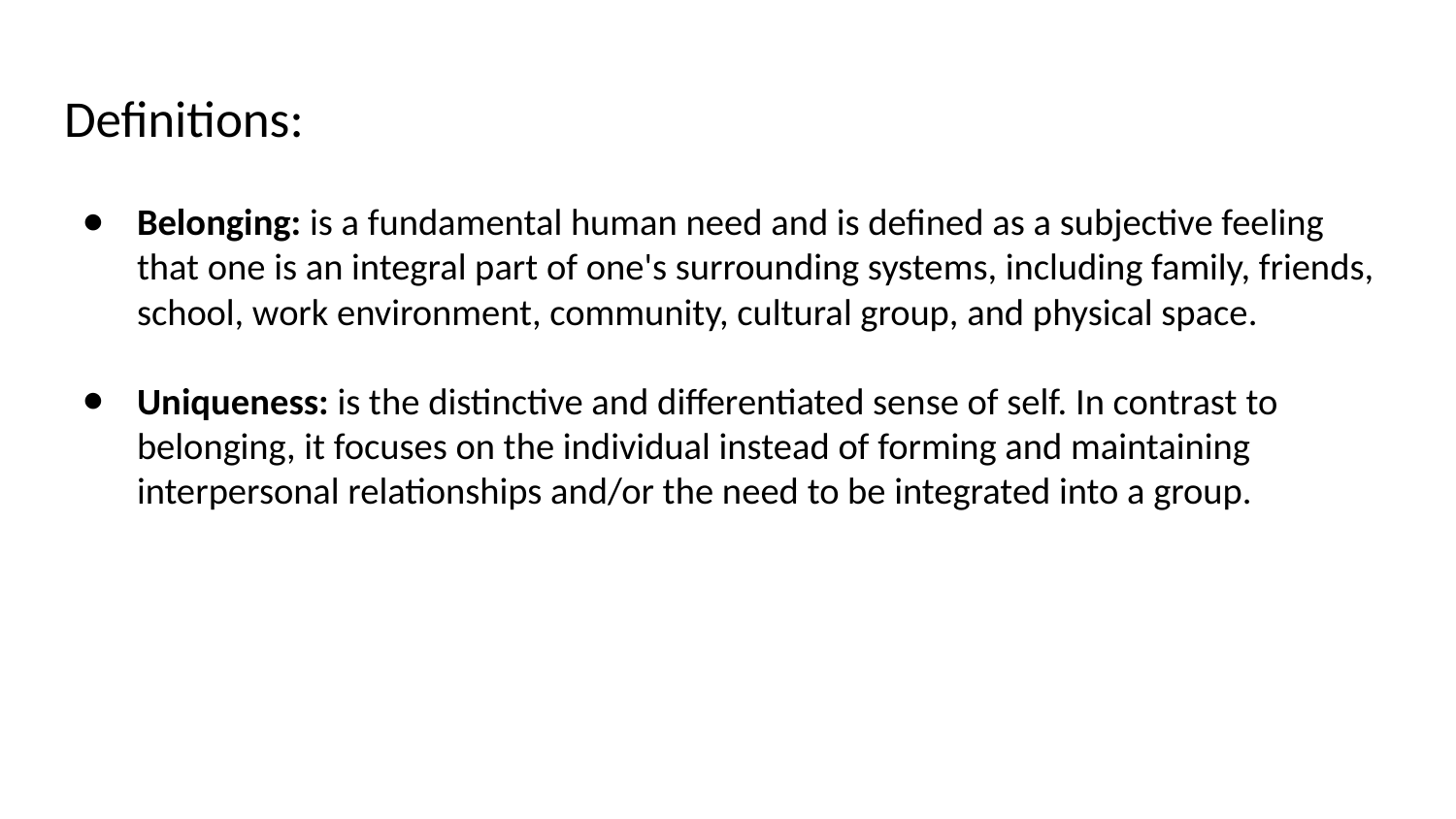

# Definitions:
Belonging: is a fundamental human need and is defined as a subjective feeling that one is an integral part of one's surrounding systems, including family, friends, school, work environment, community, cultural group, and physical space.
Uniqueness: is the distinctive and differentiated sense of self. In contrast to belonging, it focuses on the individual instead of forming and maintaining interpersonal relationships and/or the need to be integrated into a group.

## Slide 6
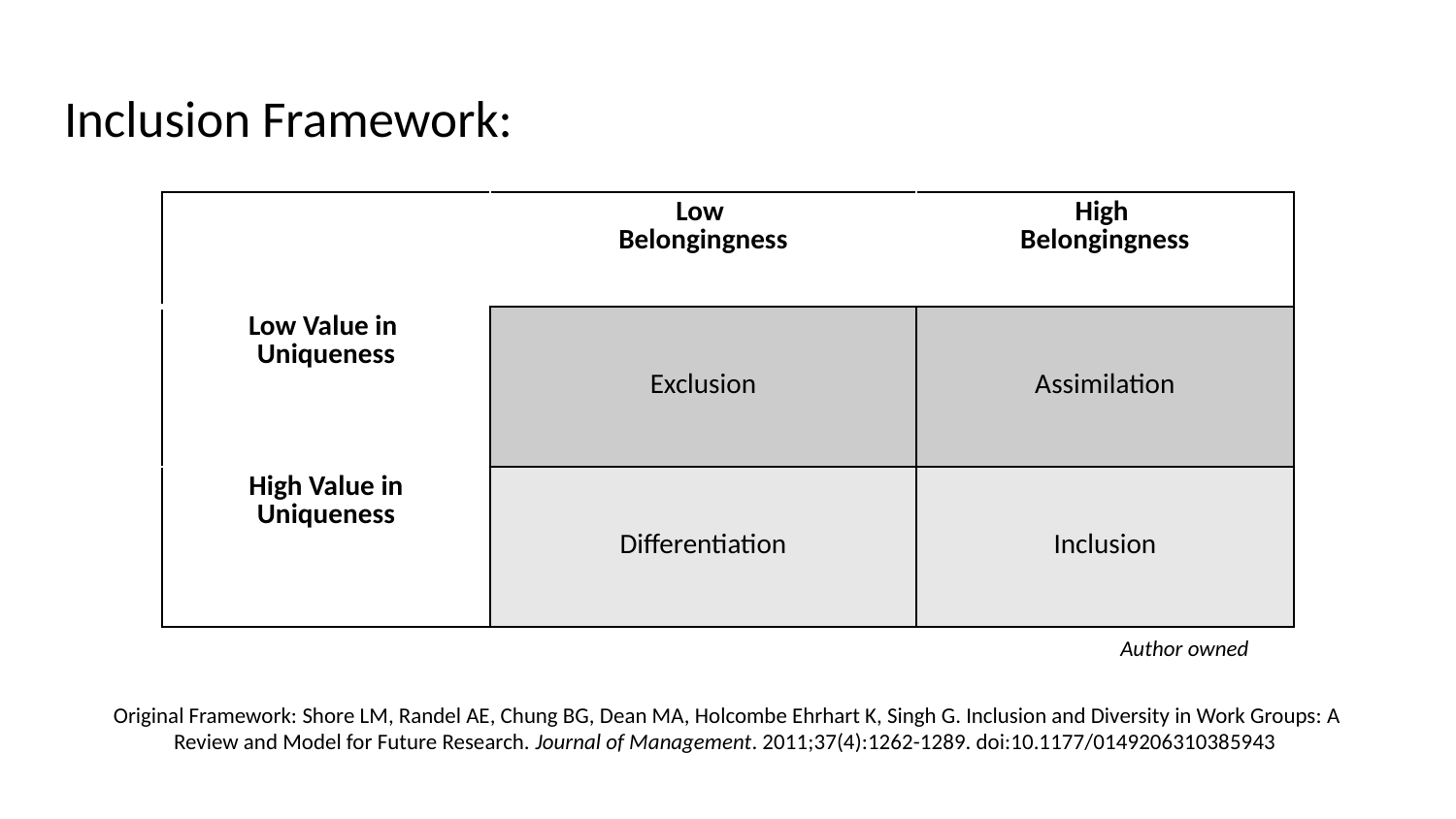

# Inclusion Framework:
| | Low Belongingness | High Belongingness |
| --- | --- | --- |
| Low Value in Uniqueness | Exclusion | Assimilation |
| High Value in Uniqueness | Differentiation | Inclusion |
Author owned
Original Framework: Shore LM, Randel AE, Chung BG, Dean MA, Holcombe Ehrhart K, Singh G. Inclusion and Diversity in Work Groups: A Review and Model for Future Research. Journal of Management. 2011;37(4):1262-1289. doi:10.1177/0149206310385943

## Slide 7
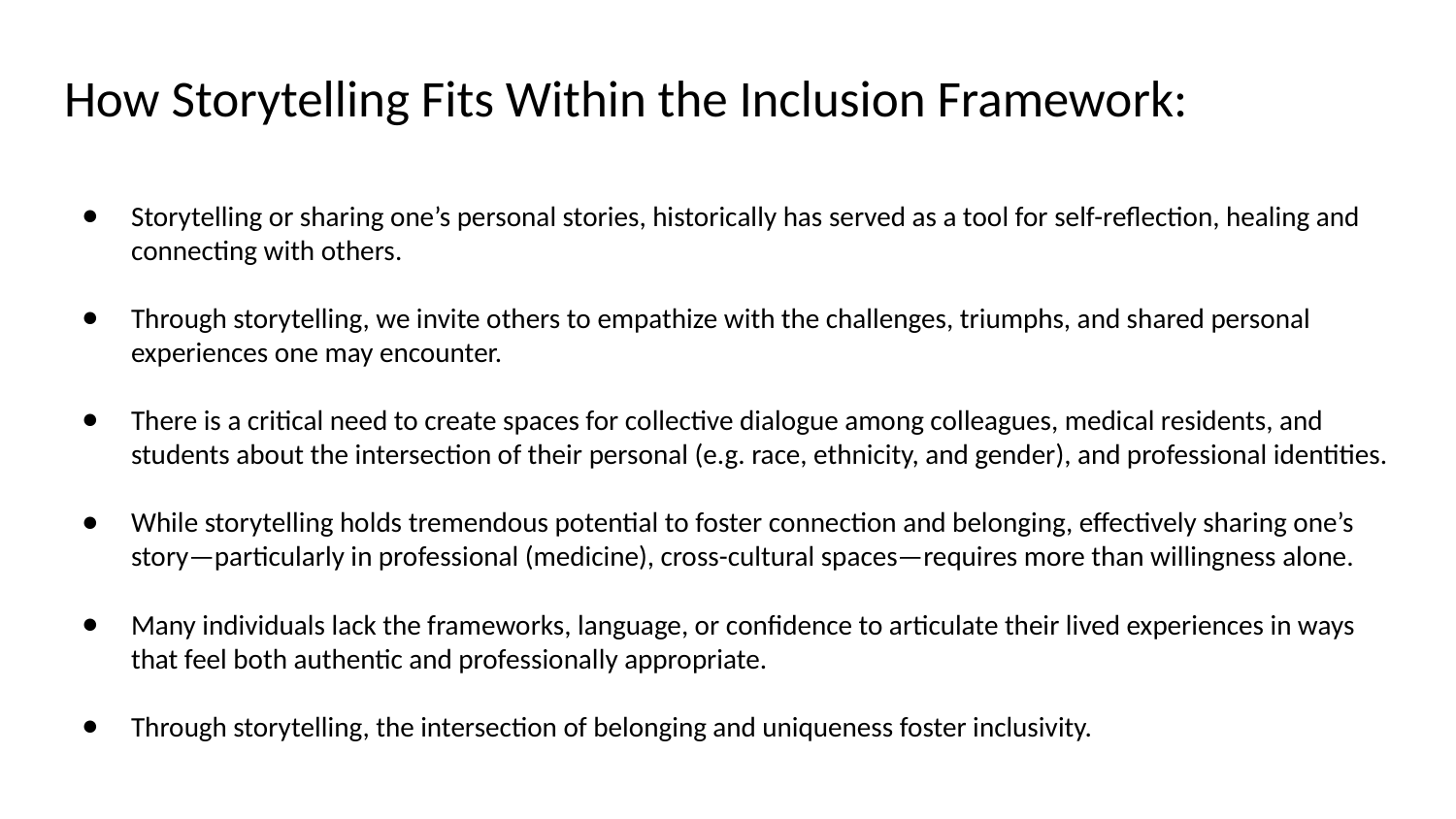

# How Storytelling Fits Within the Inclusion Framework:
Storytelling or sharing one’s personal stories, historically has served as a tool for self-reflection, healing and connecting with others.
Through storytelling, we invite others to empathize with the challenges, triumphs, and shared personal experiences one may encounter.
There is a critical need to create spaces for collective dialogue among colleagues, medical residents, and students about the intersection of their personal (e.g. race, ethnicity, and gender), and professional identities.
While storytelling holds tremendous potential to foster connection and belonging, effectively sharing one’s story—particularly in professional (medicine), cross-cultural spaces—requires more than willingness alone.
Many individuals lack the frameworks, language, or confidence to articulate their lived experiences in ways that feel both authentic and professionally appropriate.
Through storytelling, the intersection of belonging and uniqueness foster inclusivity.

## Slide 8
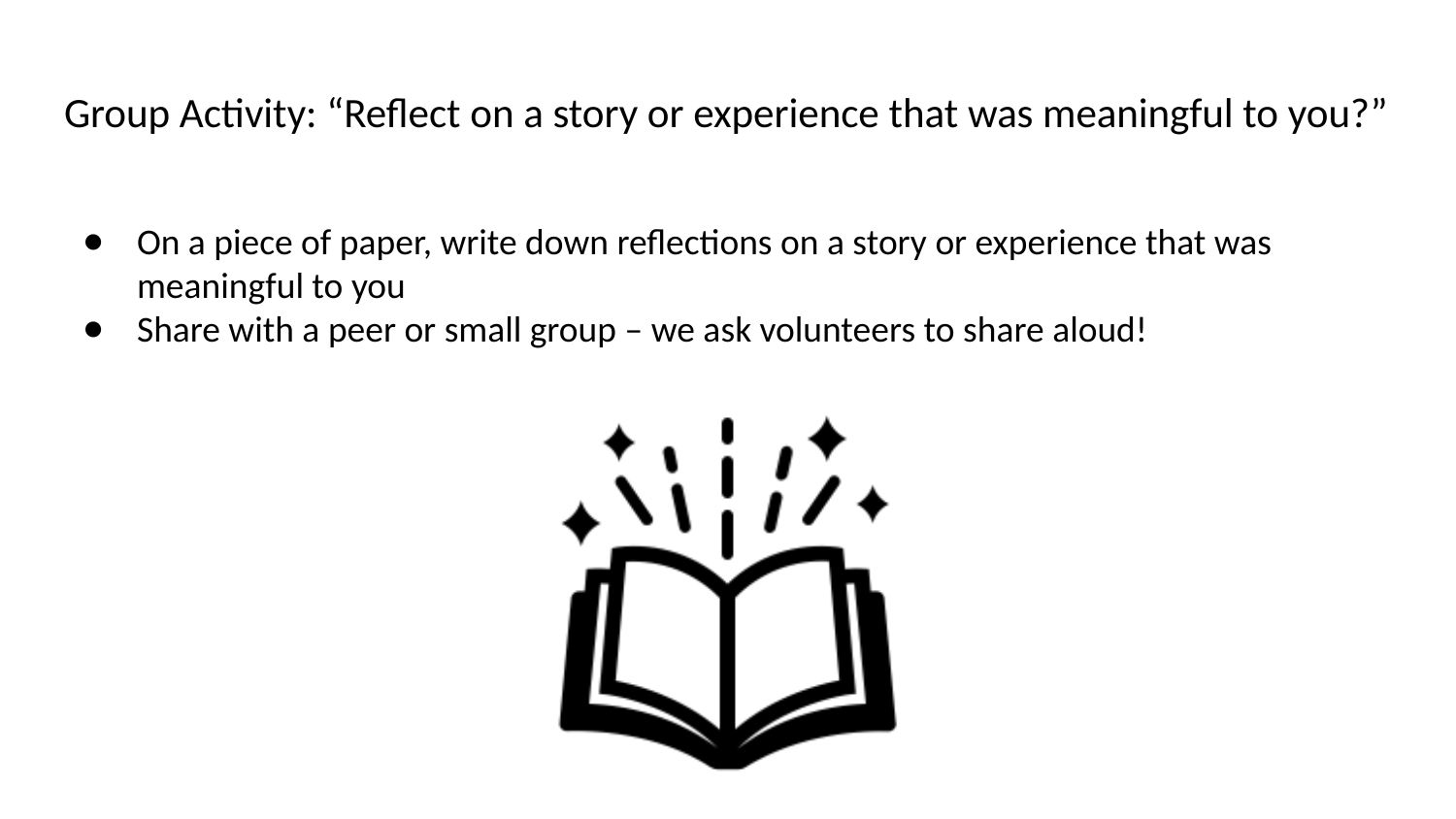

# Group Activity: “Reflect on a story or experience that was meaningful to you?”
On a piece of paper, write down reflections on a story or experience that was meaningful to you
Share with a peer or small group – we ask volunteers to share aloud!

## Slide 9
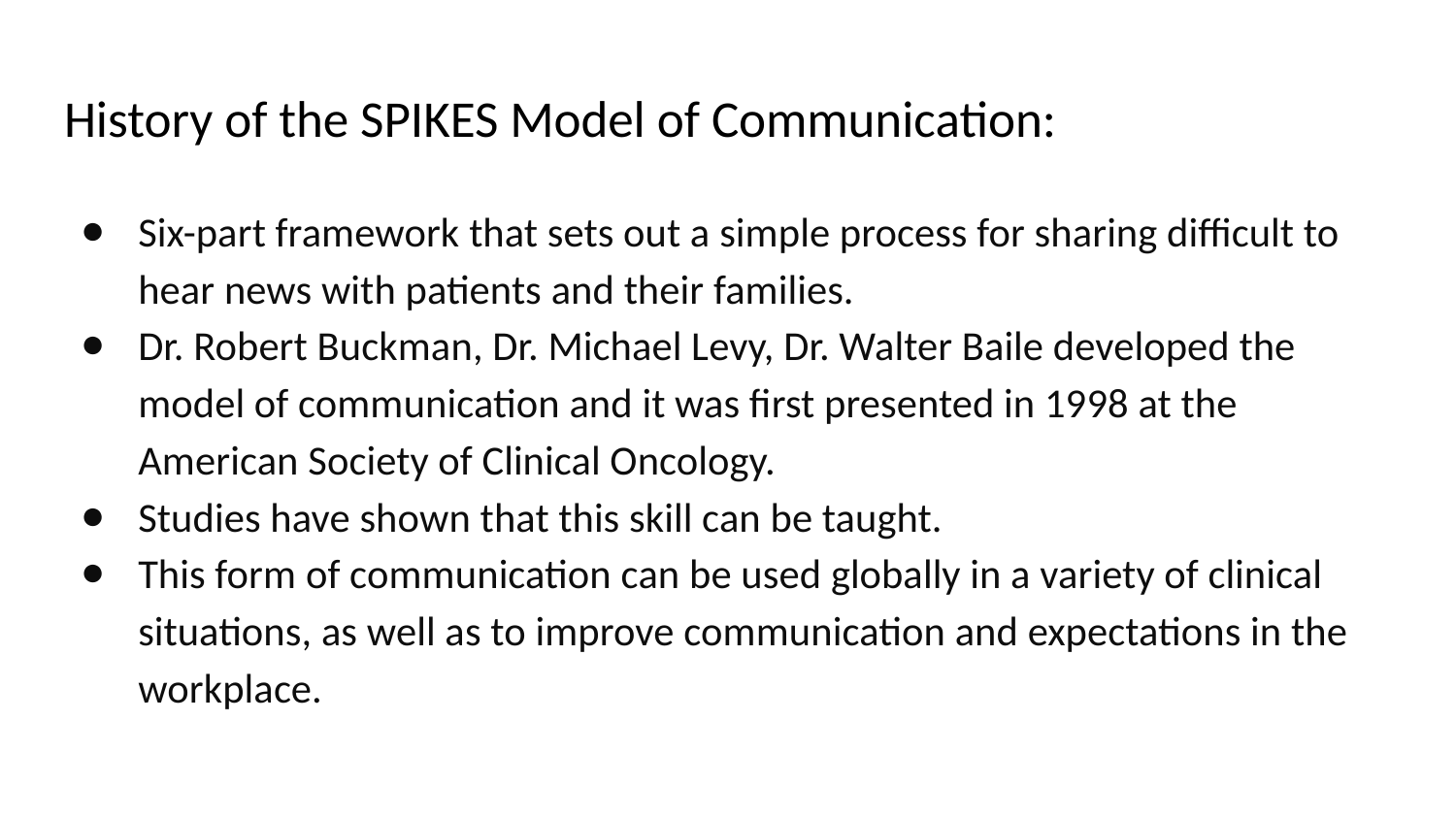

# History of the SPIKES Model of Communication:
Six-part framework that sets out a simple process for sharing difficult to hear news with patients and their families.
Dr. Robert Buckman, Dr. Michael Levy, Dr. Walter Baile developed the model of communication and it was first presented in 1998 at the American Society of Clinical Oncology.
Studies have shown that this skill can be taught.
This form of communication can be used globally in a variety of clinical situations, as well as to improve communication and expectations in the workplace.

## Slide 10
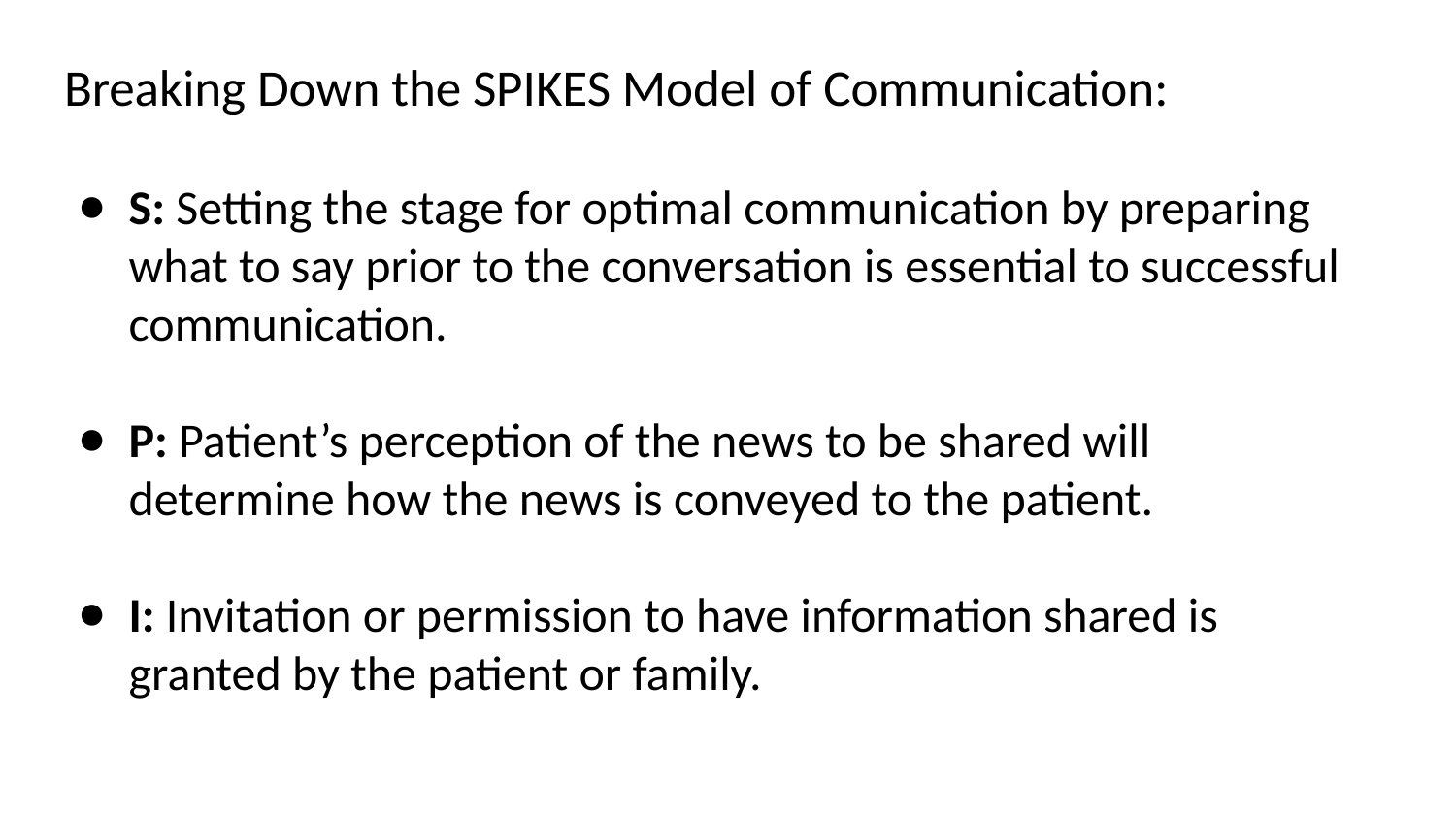

# Breaking Down the SPIKES Model of Communication:
S: Setting the stage for optimal communication by preparing what to say prior to the conversation is essential to successful communication.
P: Patient’s perception of the news to be shared will determine how the news is conveyed to the patient.
I: Invitation or permission to have information shared is granted by the patient or family.

## Slide 11
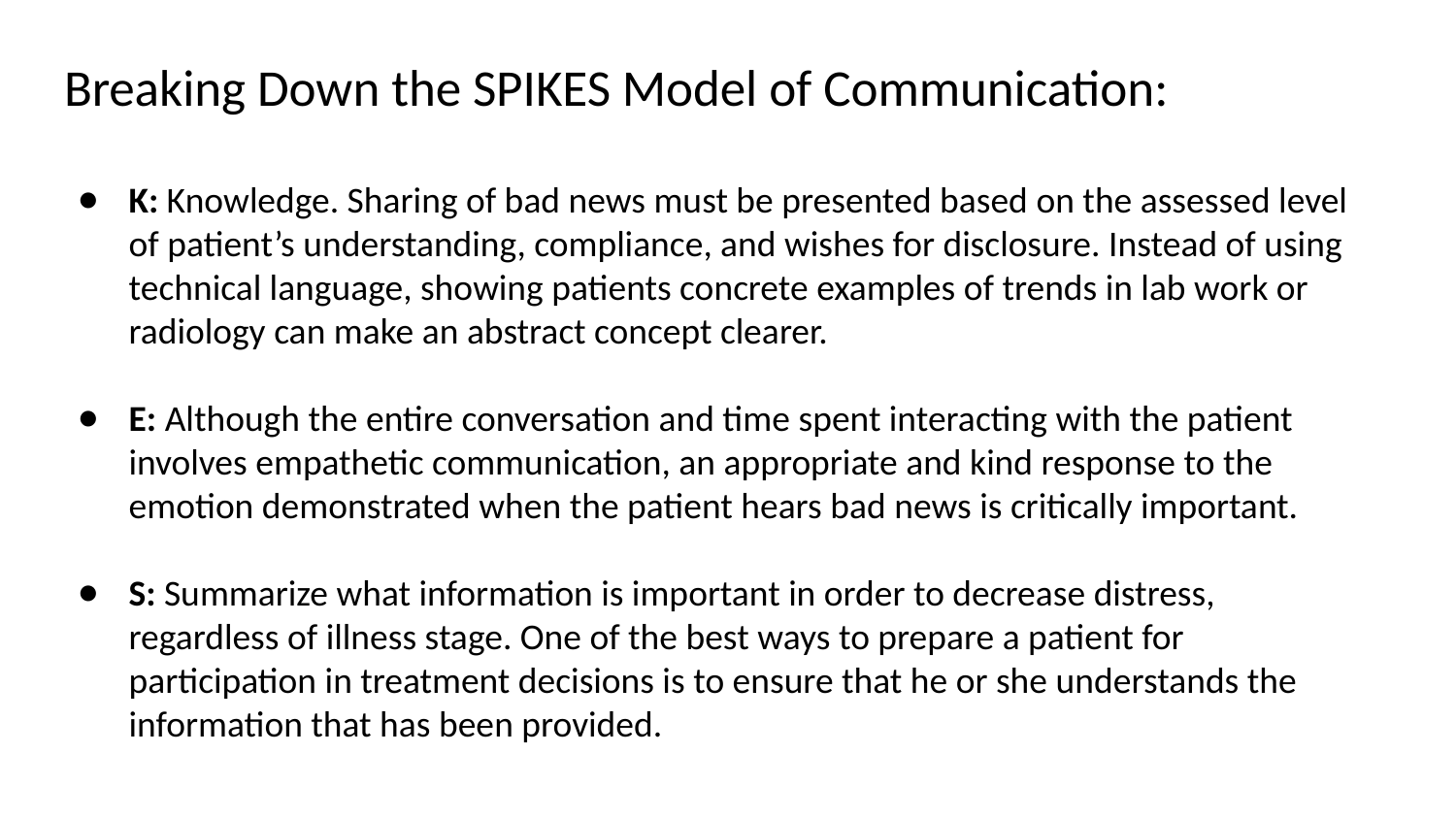

# Breaking Down the SPIKES Model of Communication:
K: Knowledge. Sharing of bad news must be presented based on the assessed level of patient’s understanding, compliance, and wishes for disclosure. Instead of using technical language, showing patients concrete examples of trends in lab work or radiology can make an abstract concept clearer.
E: Although the entire conversation and time spent interacting with the patient involves empathetic communication, an appropriate and kind response to the emotion demonstrated when the patient hears bad news is critically important.
S: Summarize what information is important in order to decrease distress, regardless of illness stage. One of the best ways to prepare a patient for participation in treatment decisions is to ensure that he or she understands the information that has been provided.

## Slide 12
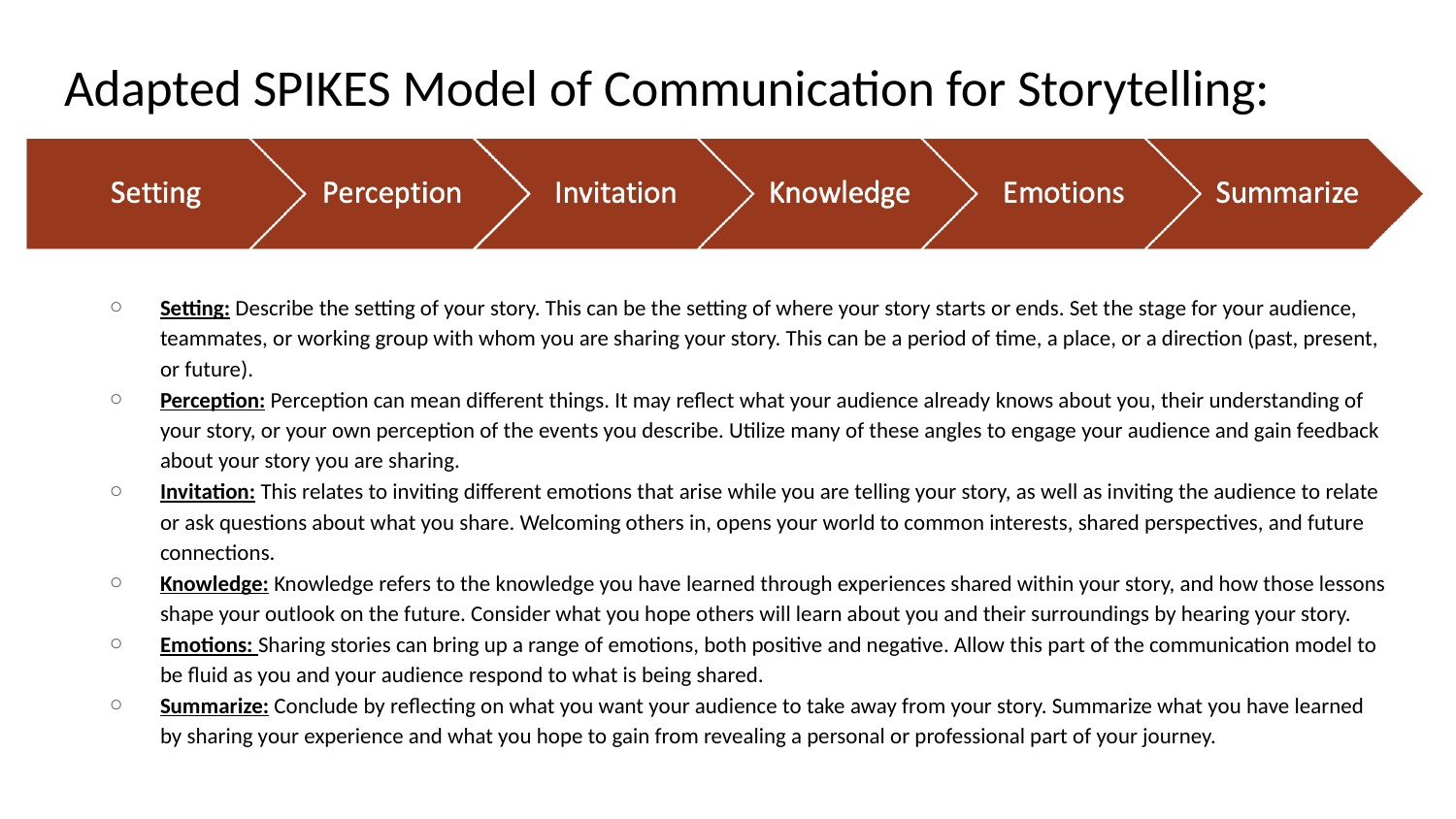

# Adapted SPIKES Model of Communication for Storytelling:
Setting: Describe the setting of your story. This can be the setting of where your story starts or ends. Set the stage for your audience, teammates, or working group with whom you are sharing your story. This can be a period of time, a place, or a direction (past, present, or future).
Perception: Perception can mean different things. It may reflect what your audience already knows about you, their understanding of your story, or your own perception of the events you describe. Utilize many of these angles to engage your audience and gain feedback about your story you are sharing.
Invitation: This relates to inviting different emotions that arise while you are telling your story, as well as inviting the audience to relate or ask questions about what you share. Welcoming others in, opens your world to common interests, shared perspectives, and future connections.
Knowledge: Knowledge refers to the knowledge you have learned through experiences shared within your story, and how those lessons shape your outlook on the future. Consider what you hope others will learn about you and their surroundings by hearing your story.
Emotions: Sharing stories can bring up a range of emotions, both positive and negative. Allow this part of the communication model to be fluid as you and your audience respond to what is being shared.
Summarize: Conclude by reflecting on what you want your audience to take away from your story. Summarize what you have learned by sharing your experience and what you hope to gain from revealing a personal or professional part of your journey.

## Slide 13
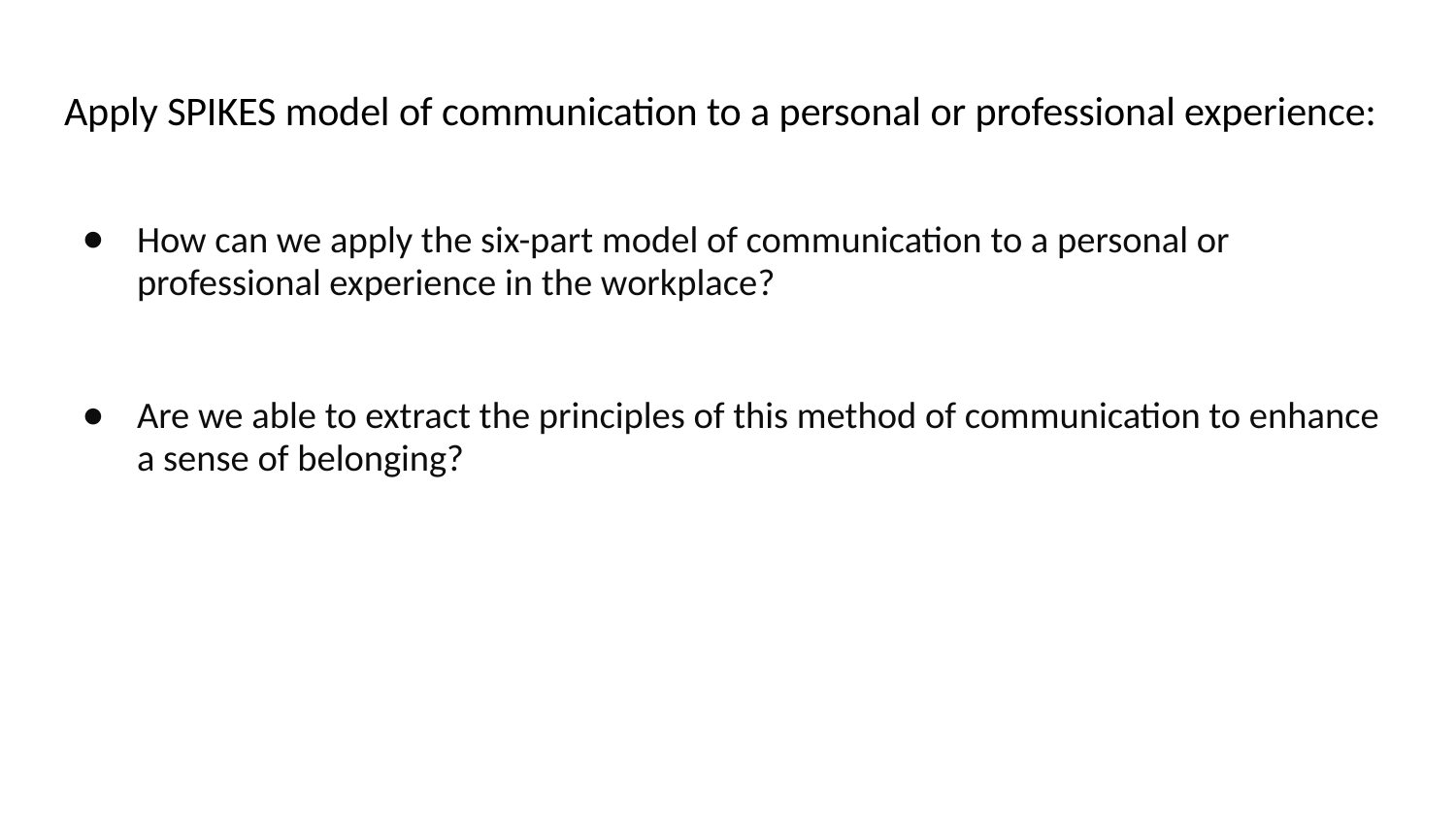

# Apply SPIKES model of communication to a personal or professional experience:
How can we apply the six-part model of communication to a personal or professional experience in the workplace?
Are we able to extract the principles of this method of communication to enhance a sense of belonging?

## Slide 14
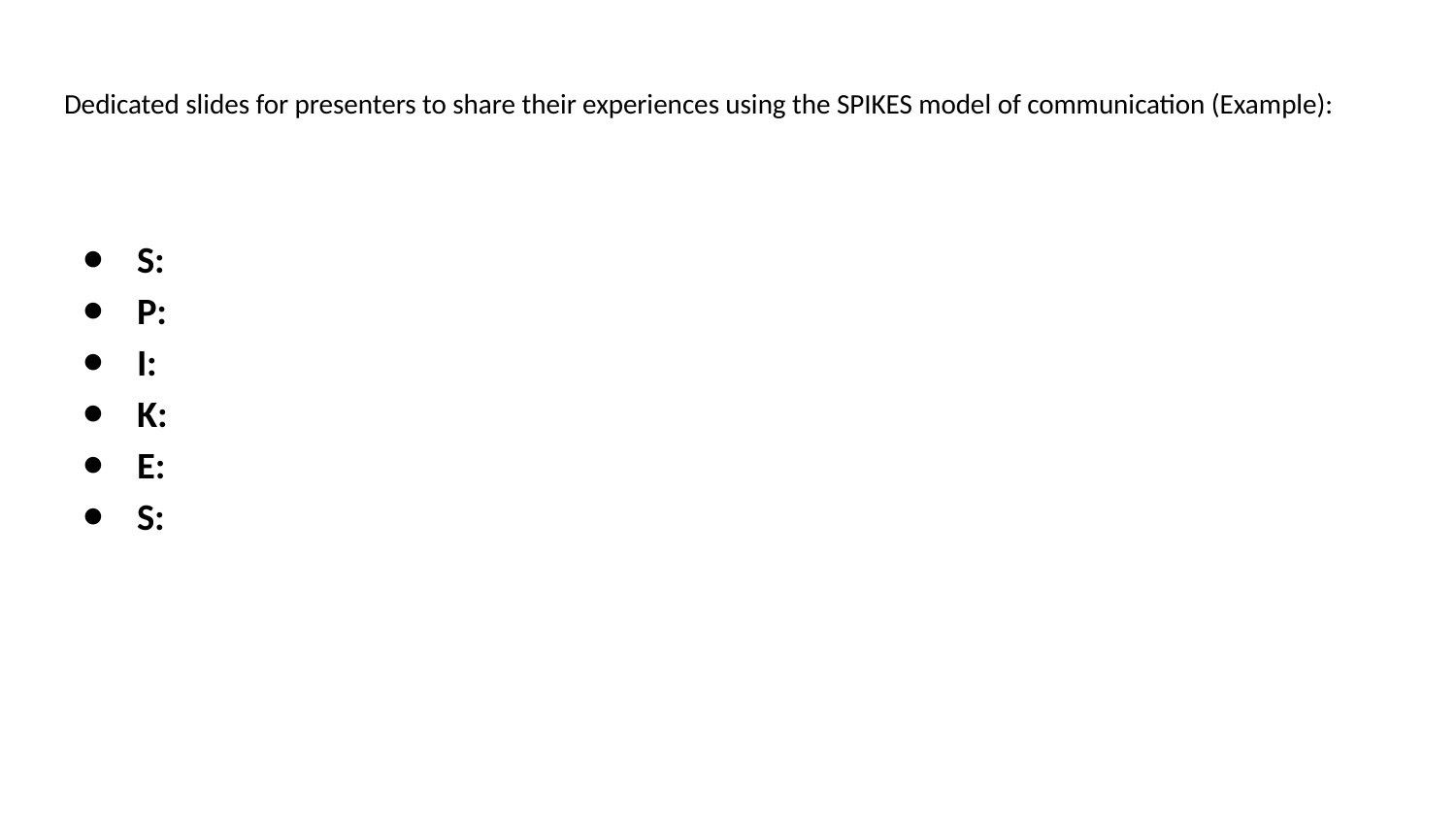

# Dedicated slides for presenters to share their experiences using the SPIKES model of communication (Example):
S:
P:
I:
K:
E:
S:

## Slide 15
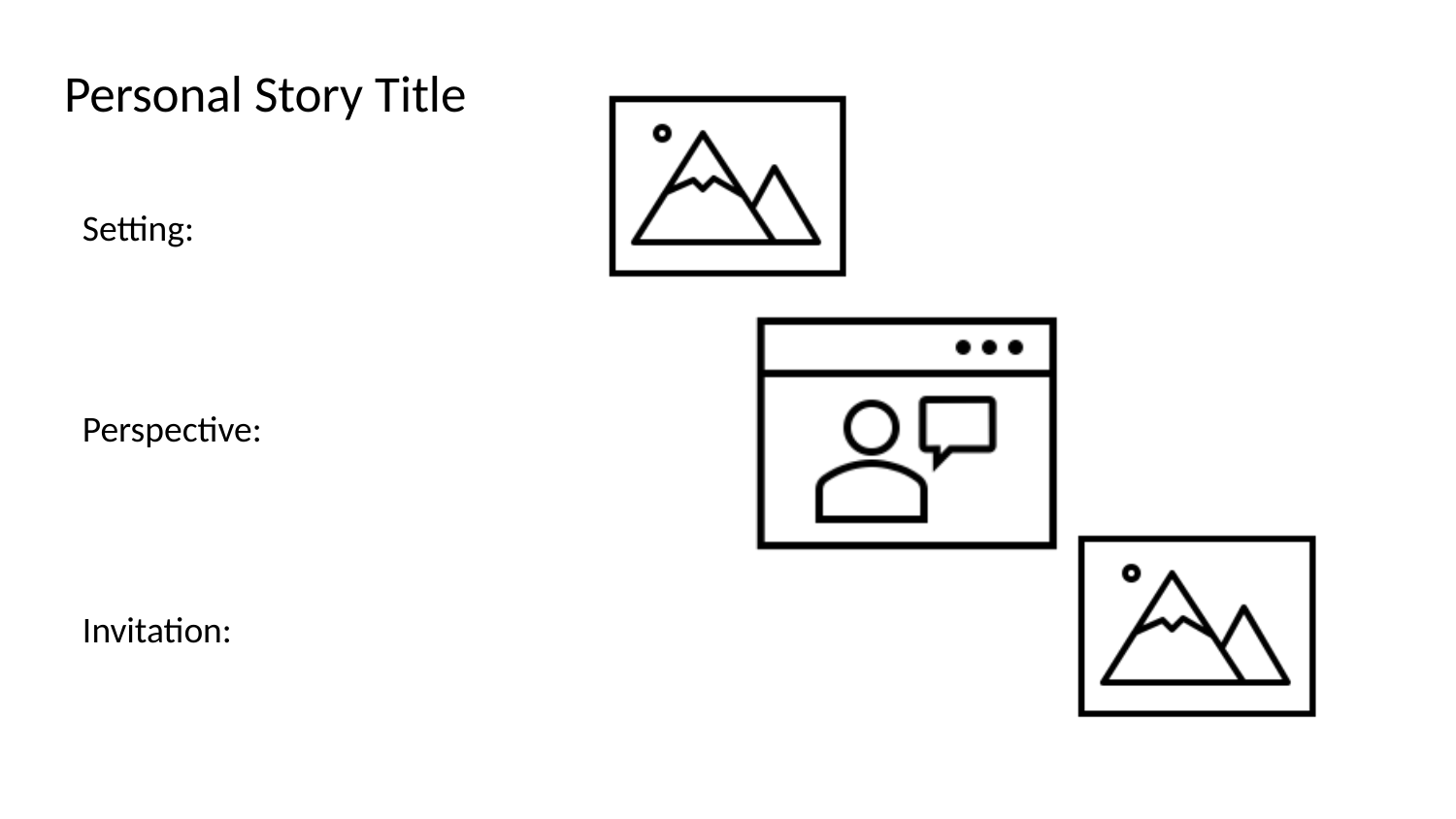

# Personal Story Title
Setting:
Perspective:
Invitation:

## Slide 16
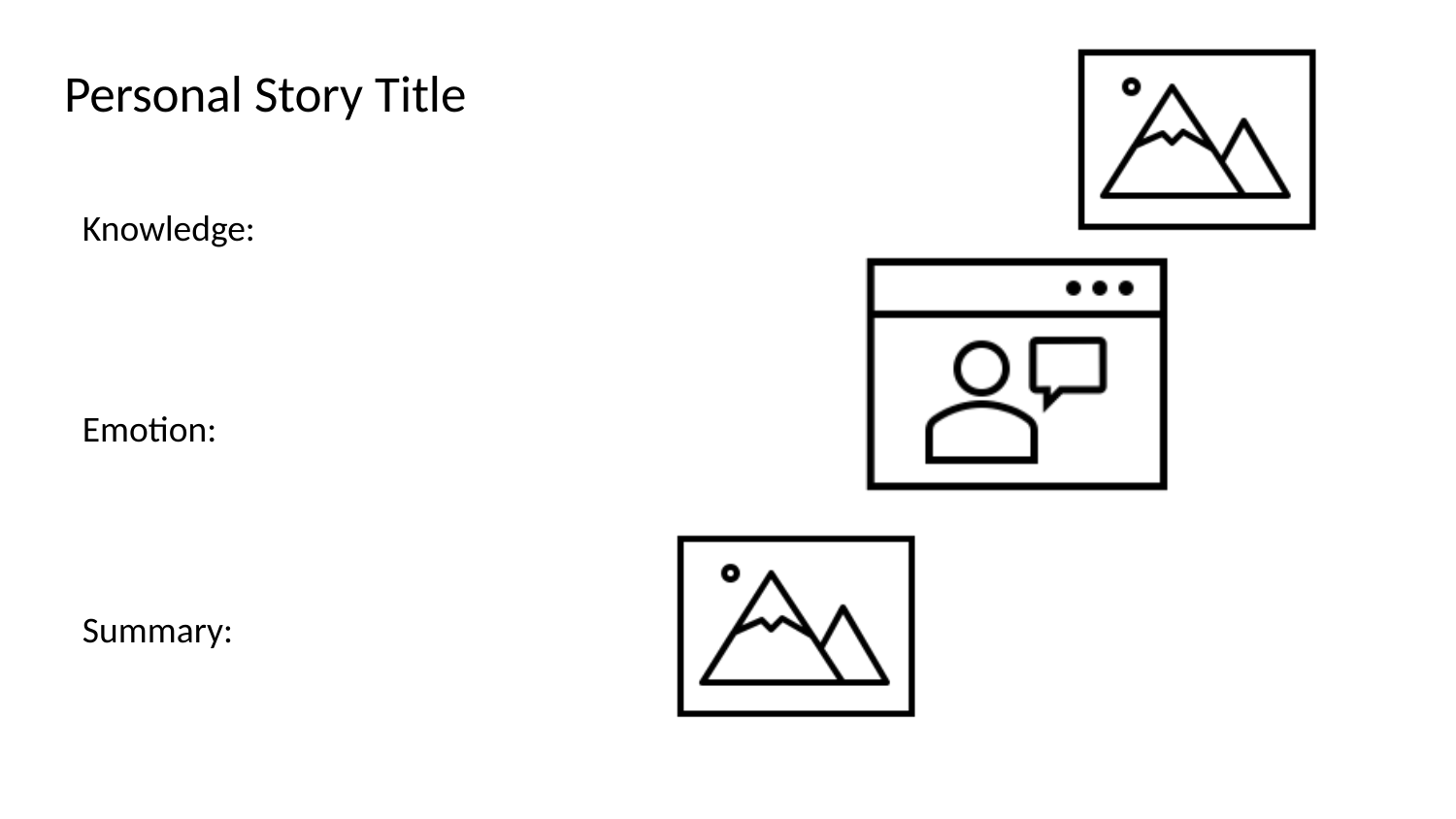

# Personal Story Title
Knowledge:
Emotion:
Summary:

## Slide 17
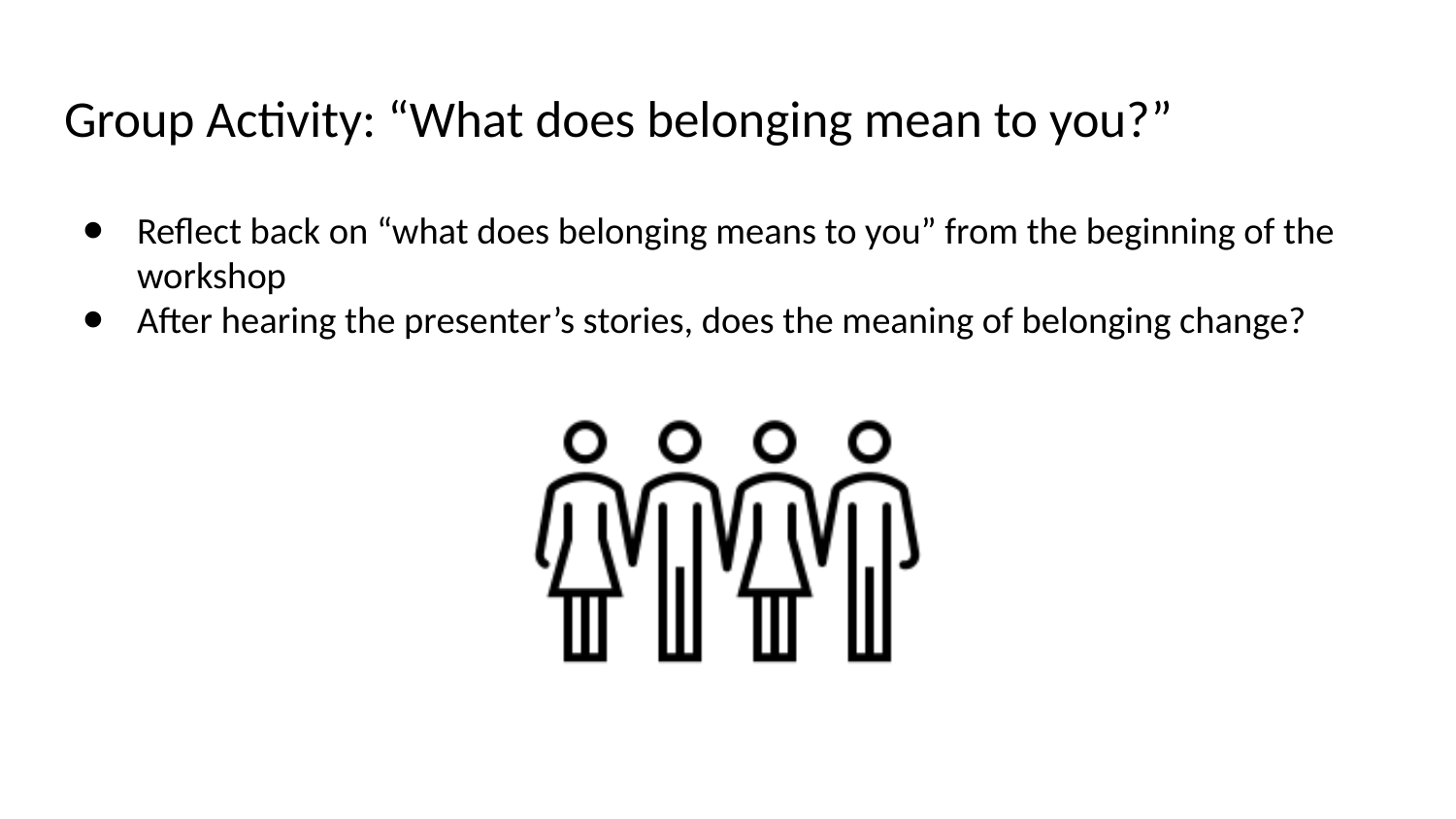

# Group Activity: “What does belonging mean to you?”
Reflect back on “what does belonging means to you” from the beginning of the workshop
After hearing the presenter’s stories, does the meaning of belonging change?

## Slide 18
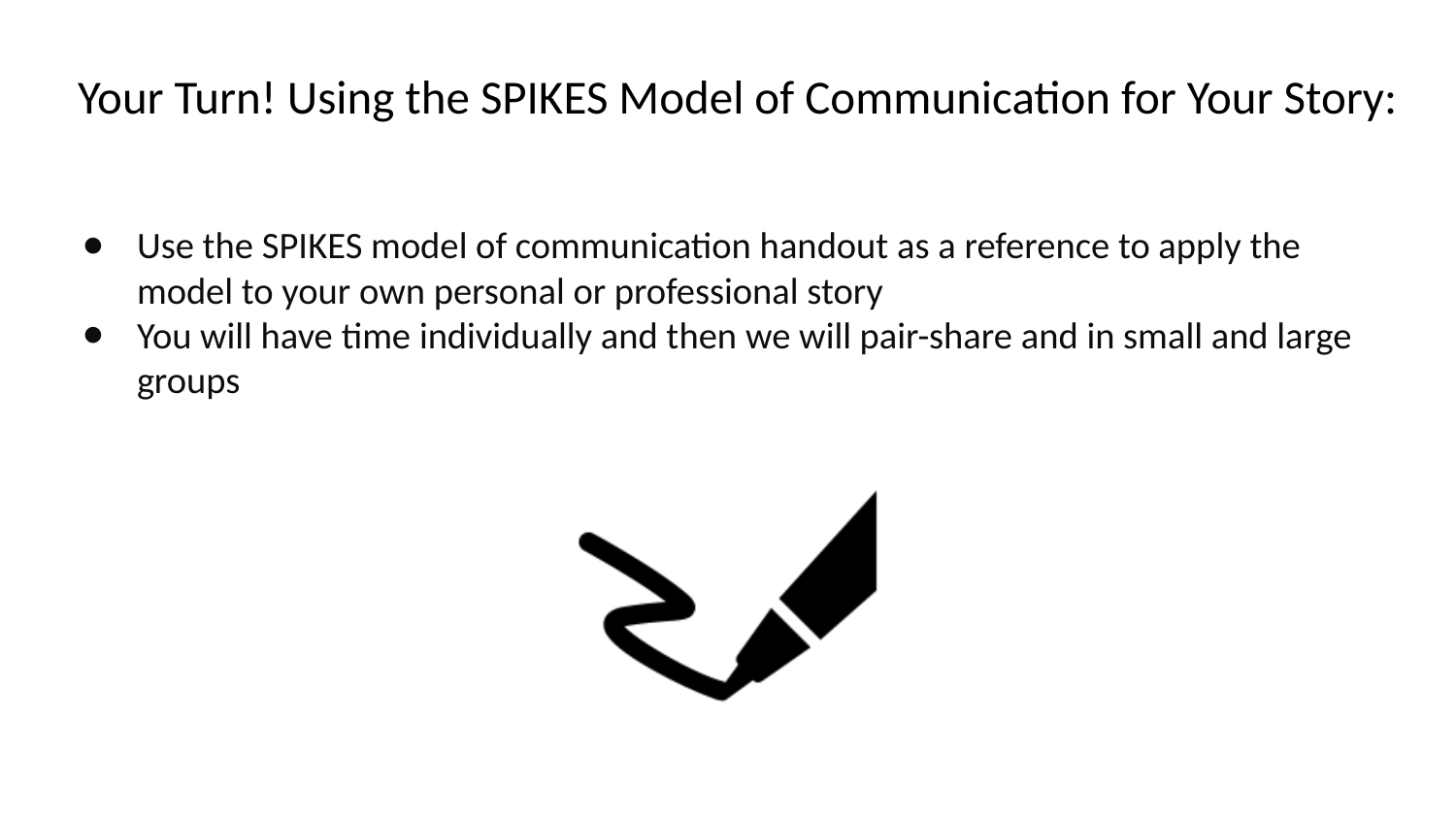

# Your Turn! Using the SPIKES Model of Communication for Your Story:
Use the SPIKES model of communication handout as a reference to apply the model to your own personal or professional story
You will have time individually and then we will pair-share and in small and large groups

## Slide 19
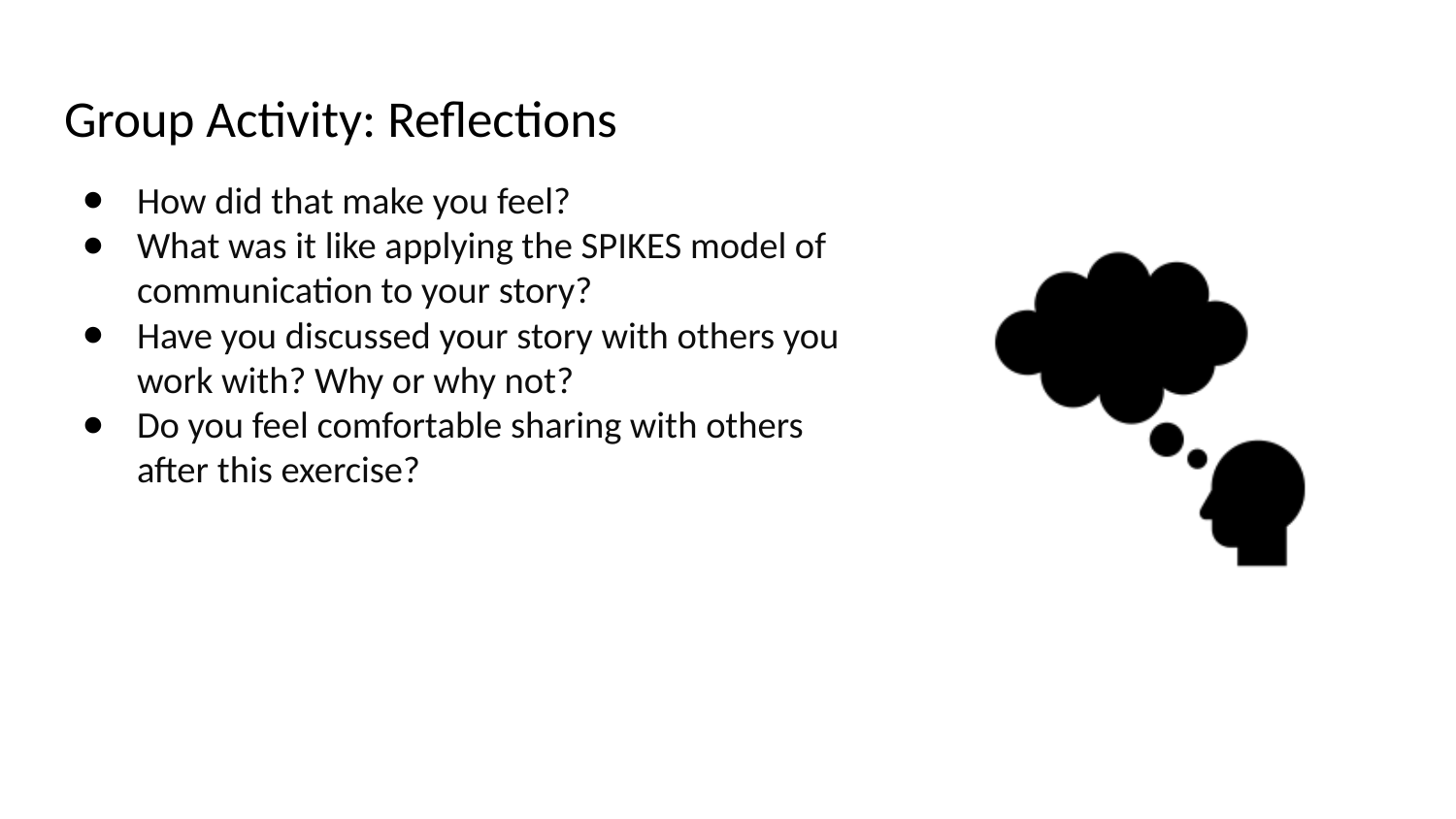

# Group Activity: Reflections
How did that make you feel?
What was it like applying the SPIKES model of communication to your story?
Have you discussed your story with others you work with? Why or why not?
Do you feel comfortable sharing with others after this exercise?

## Slide 20
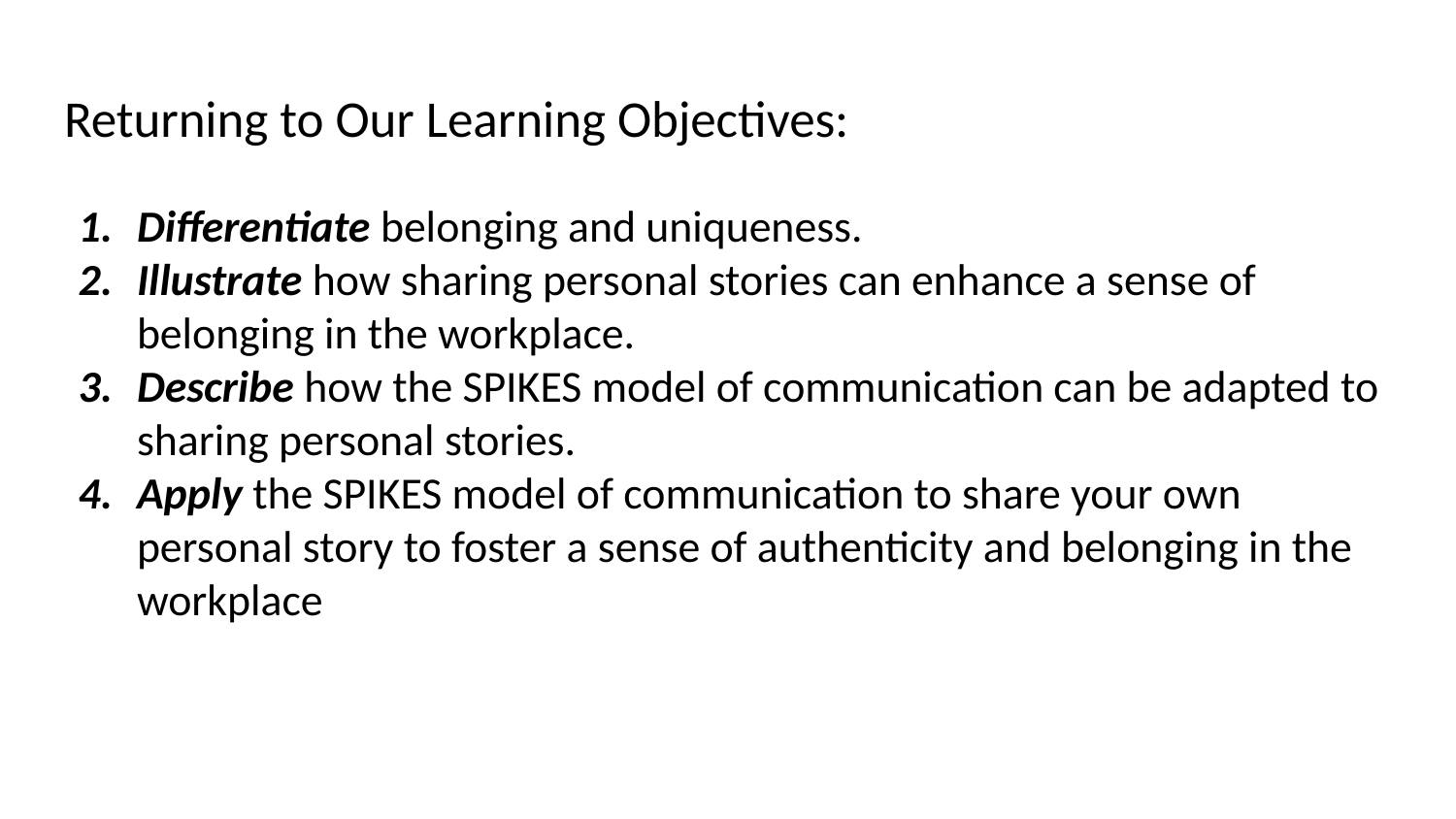

# Returning to Our Learning Objectives:
Differentiate belonging and uniqueness.
Illustrate how sharing personal stories can enhance a sense of belonging in the workplace.
Describe how the SPIKES model of communication can be adapted to sharing personal stories.
Apply the SPIKES model of communication to share your own personal story to foster a sense of authenticity and belonging in the workplace

## Slide 21
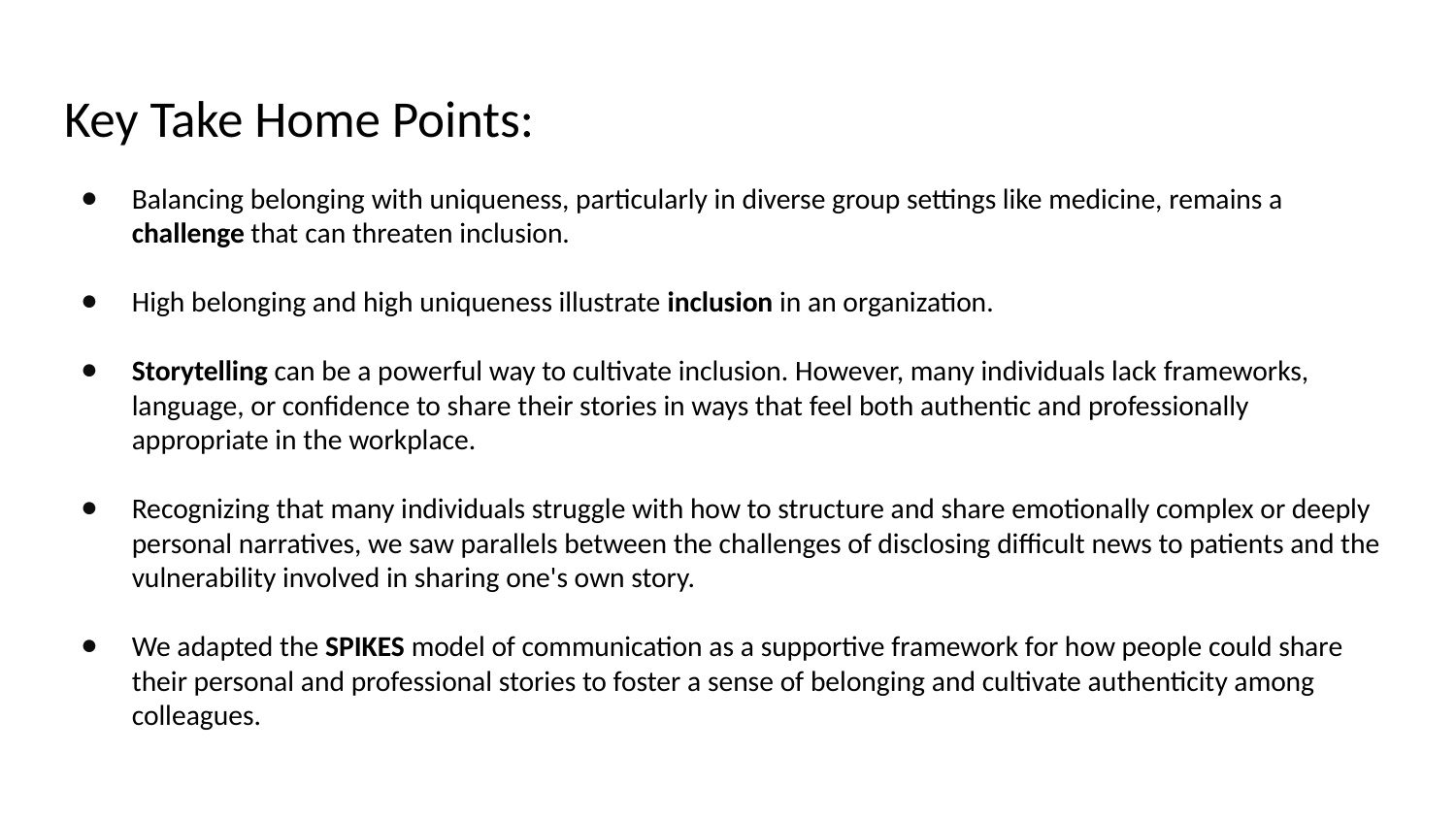

# Key Take Home Points:
Balancing belonging with uniqueness, particularly in diverse group settings like medicine, remains a challenge that can threaten inclusion.
High belonging and high uniqueness illustrate inclusion in an organization.
Storytelling can be a powerful way to cultivate inclusion. However, many individuals lack frameworks, language, or confidence to share their stories in ways that feel both authentic and professionally appropriate in the workplace.
Recognizing that many individuals struggle with how to structure and share emotionally complex or deeply personal narratives, we saw parallels between the challenges of disclosing difficult news to patients and the vulnerability involved in sharing one's own story.
We adapted the SPIKES model of communication as a supportive framework for how people could share their personal and professional stories to foster a sense of belonging and cultivate authenticity among colleagues.

## Slide 22
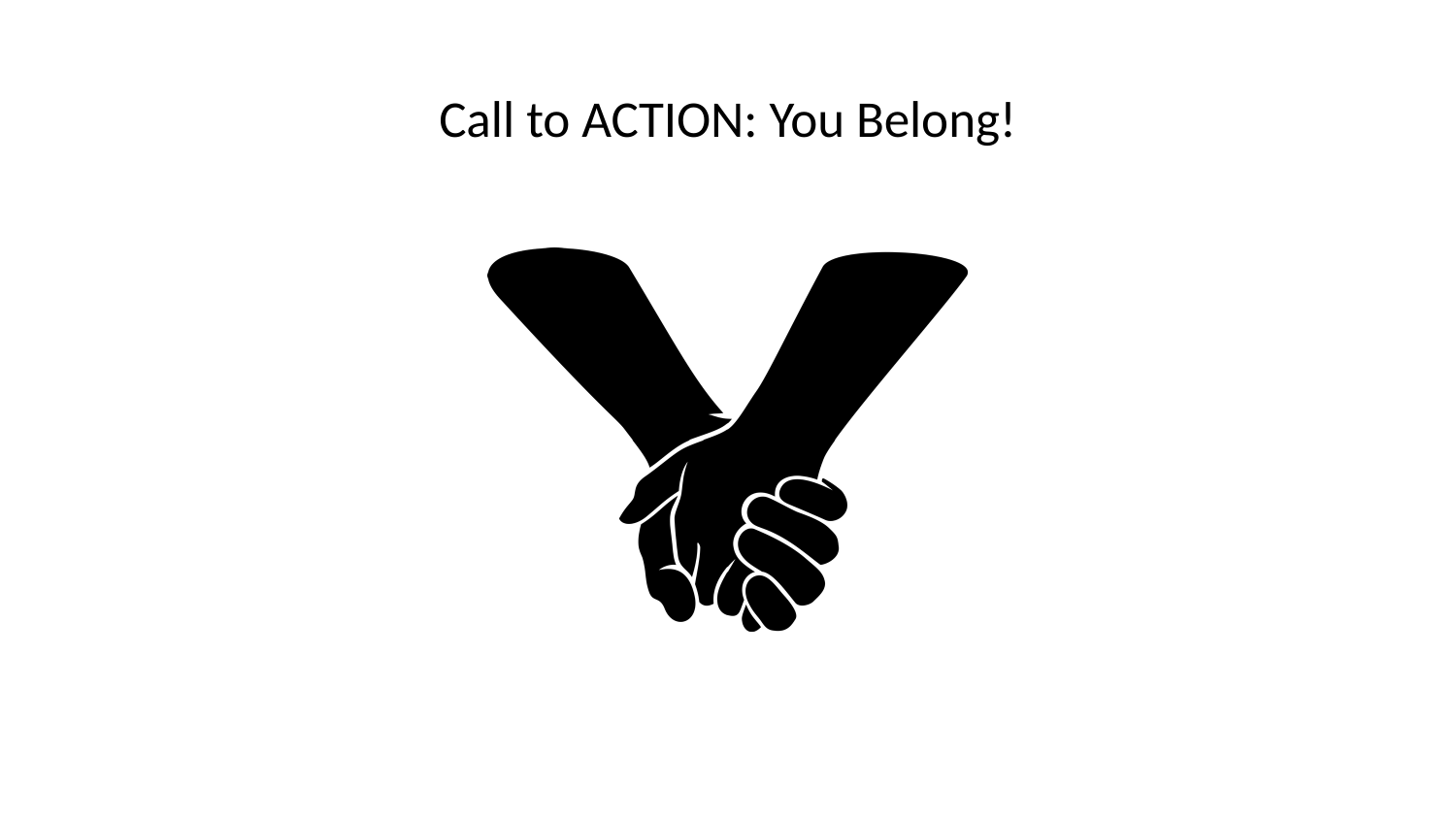

# Call to ACTION: You Belong!

## Slide 23
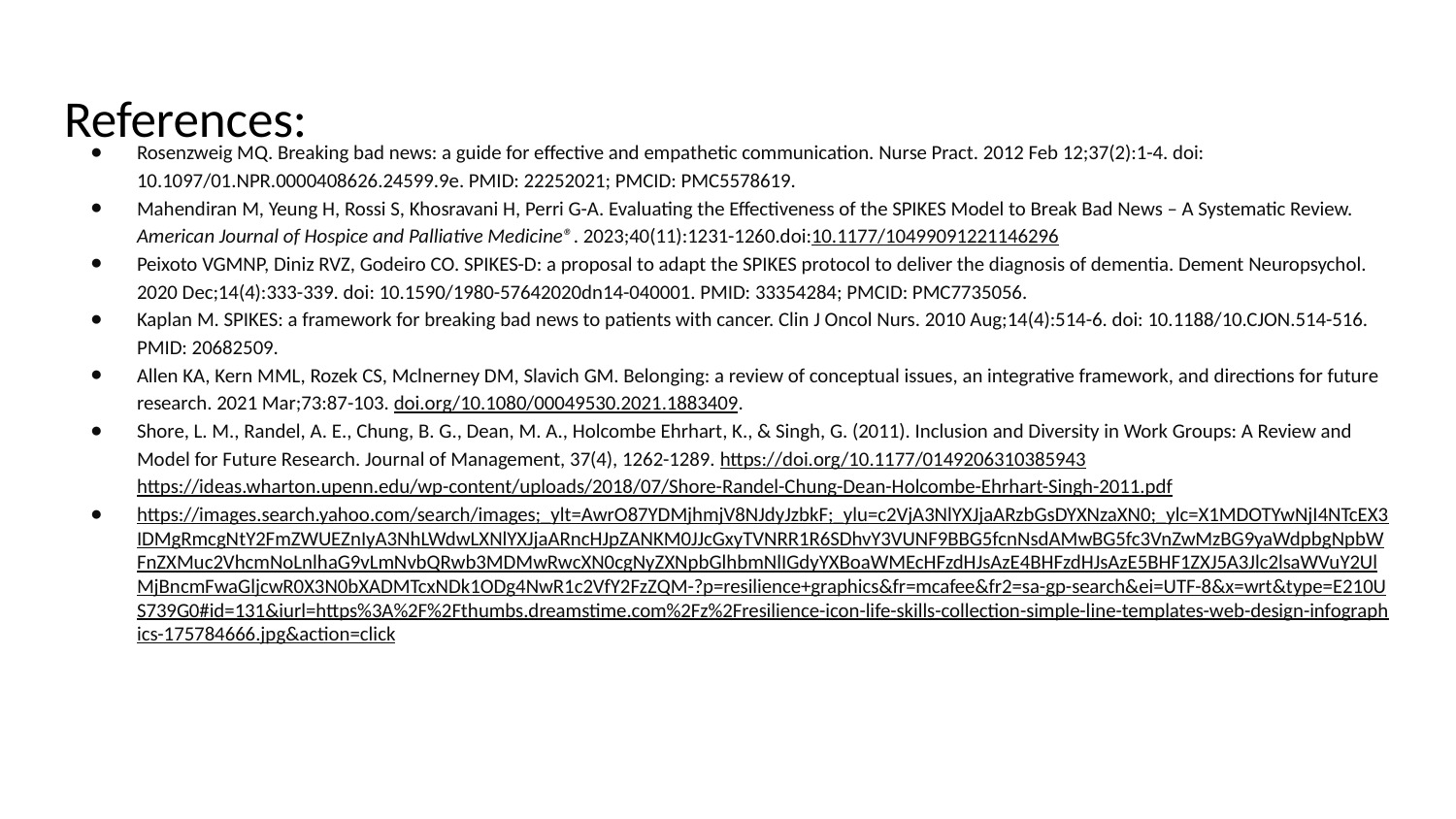

# References:
Rosenzweig MQ. Breaking bad news: a guide for effective and empathetic communication. Nurse Pract. 2012 Feb 12;37(2):1-4. doi: 10.1097/01.NPR.0000408626.24599.9e. PMID: 22252021; PMCID: PMC5578619.
Mahendiran M, Yeung H, Rossi S, Khosravani H, Perri G-A. Evaluating the Effectiveness of the SPIKES Model to Break Bad News – A Systematic Review. American Journal of Hospice and Palliative Medicine®. 2023;40(11):1231-1260.doi:10.1177/10499091221146296
Peixoto VGMNP, Diniz RVZ, Godeiro CO. SPIKES-D: a proposal to adapt the SPIKES protocol to deliver the diagnosis of dementia. Dement Neuropsychol. 2020 Dec;14(4):333-339. doi: 10.1590/1980-57642020dn14-040001. PMID: 33354284; PMCID: PMC7735056.
Kaplan M. SPIKES: a framework for breaking bad news to patients with cancer. Clin J Oncol Nurs. 2010 Aug;14(4):514-6. doi: 10.1188/10.CJON.514-516. PMID: 20682509.
Allen KA, Kern MML, Rozek CS, Mclnerney DM, Slavich GM. Belonging: a review of conceptual issues, an integrative framework, and directions for future research. 2021 Mar;73:87-103. doi.org/10.1080/00049530.2021.1883409.
Shore, L. M., Randel, A. E., Chung, B. G., Dean, M. A., Holcombe Ehrhart, K., & Singh, G. (2011). Inclusion and Diversity in Work Groups: A Review and Model for Future Research. Journal of Management, 37(4), 1262-1289. https://doi.org/10.1177/0149206310385943 https://ideas.wharton.upenn.edu/wp-content/uploads/2018/07/Shore-Randel-Chung-Dean-Holcombe-Ehrhart-Singh-2011.pdf
https://images.search.yahoo.com/search/images;_ylt=AwrO87YDMjhmjV8NJdyJzbkF;_ylu=c2VjA3NlYXJjaARzbGsDYXNzaXN0;_ylc=X1MDOTYwNjI4NTcEX3IDMgRmcgNtY2FmZWUEZnIyA3NhLWdwLXNlYXJjaARncHJpZANKM0JJcGxyTVNRR1R6SDhvY3VUNF9BBG5fcnNsdAMwBG5fc3VnZwMzBG9yaWdpbgNpbWFnZXMuc2VhcmNoLnlhaG9vLmNvbQRwb3MDMwRwcXN0cgNyZXNpbGlhbmNlIGdyYXBoaWMEcHFzdHJsAzE4BHFzdHJsAzE5BHF1ZXJ5A3Jlc2lsaWVuY2UlMjBncmFwaGljcwR0X3N0bXADMTcxNDk1ODg4NwR1c2VfY2FzZQM-?p=resilience+graphics&fr=mcafee&fr2=sa-gp-search&ei=UTF-8&x=wrt&type=E210US739G0#id=131&iurl=https%3A%2F%2Fthumbs.dreamstime.com%2Fz%2Fresilience-icon-life-skills-collection-simple-line-templates-web-design-infographics-175784666.jpg&action=click

## Slide 24
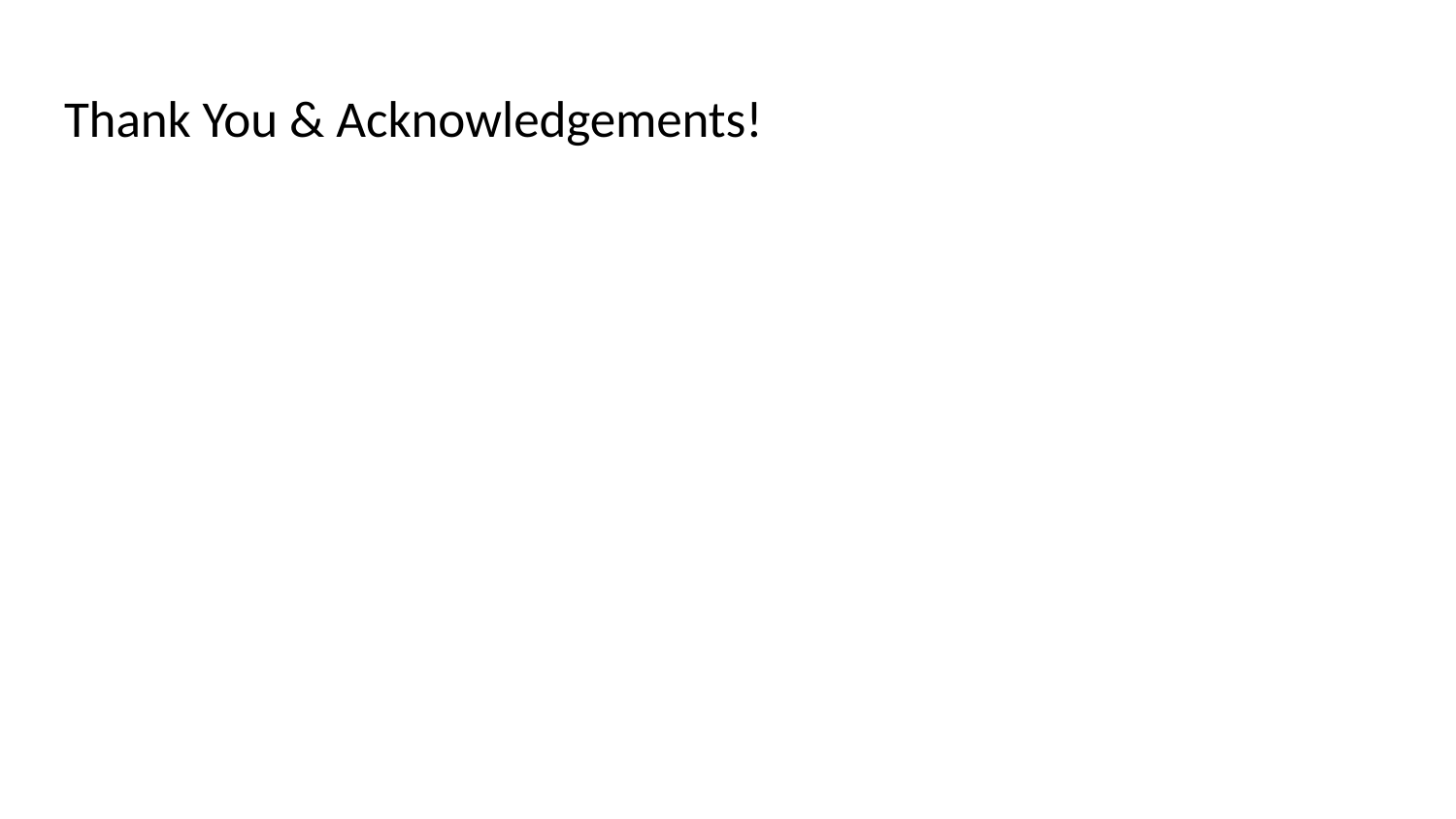

# Thank You & Acknowledgements!
