# Supplementary material for: SPIKING A Sense of Belonging: Utilizing a Communication Model to Unlock Your Story With Authenticity
Source: MedEdPORTAL. 2025 Dec 30;21:11567. doi: 10.15766/mep_2374-8265.11567 (PMC12748279; doi:10.15766/mep_2374-8265.11567)
Supplement: Supplementary file 1 — Workshop Presentation.pptxFacilitator Guide.docxHandout.docxPresentation Script.docxEvaluation Form.docx [file mep_2374-8265.11567-s001.zip › B. Facilitator Guide.docx]

**SPIKING A Sense of Belonging: Utilizing a Communication Model to Unlock Your Story with Authenticity**

*Facilitator Guide*

**Facilitator Guide Content:**

- Educational Objectives
- Pre-Reading Assignments for Facilitators
- Supplies Needed
- Workshop Agenda

**Educational Objectives:**

1. ***Differentiate*** belonging and uniqueness.
2. ***Illustrate*** how sharing personal stories can enhance a sense of belonging in the workplace.
3. ***Describe*** how the SPIKES model of communication can be adapted to sharing personal stories.
4. ***Apply*** the SPIKES model of communication to share your own personal story to foster a sense of authenticity and belonging in the workplace

**Pre-Reading Assignments for Facilitators:**

1. Shore LM, Randel AE, Chung BG, Dean MA, Holcombe Ehrhart K, Singh G: Inclusion and Diversity in Work Groups: A Review and Model for Future Research. J Manag 2011; 37:1262–89
2. Allen K-A, Kern ML, Rozek CS, McInerney DM, Slavich GM: Belonging: a review of conceptual issues, an integrative framework, and directions for future research. Aust J Psychol 2021; 73:87–102
3. Baile WF, Buckman R, Lenzi R, Glober G, Beale EA, Kudelka AP: SPIKES—A Six-Step Protocol for Delivering Bad News: Application to the Patient with Cancer. The Oncologist 2000; 5:302–11
4. Mahendiran M, Yeung H, Rossi S, Khosravani H, Perri G-A: Evaluating the Effectiveness of the SPIKES Model to Break Bad News – A Systematic Review. Am J Hosp Palliat Med 2023; 40:1231–60
5. Haggins AN: To Be Seen, Heard, and Valued: Strategies to Promote a Sense of Belonging for Women and Underrepresented in Medicine Physicians. Acad Med 2020; 95:1507–10

**Supplies Needed:**

1. PowerPoint Workshop Presentation (Appendix A)
2. Facilitator Guide (Appendix B)
3. Presentation Handout: Adapted SPIKES Model of Communication (Appendix C)
4. PowerPoint Presentation Script & References (Appendix D)
5. Post-Workshop Evaluation Form (Appendix E)

| **Workshop Agenda:** *60 Minute Version* |
| --- |
| **Introductions (2 min):**   - Introduction (1 min) - Review workshop objectives (1 min)   **Individual and Small Group Activity (5 mins):**   - Participants will be asked to write down a statement telling the audience about themselves, this can be a few words to a few sentences & to reflect on what belonging means to them (2 min) - Pair-share with a neighbor or small group and depending on time, can ask a few participants to share their reflections on belonging with the large group (3 min) |
| **Didactic - Introduction to Belonging and Uniqueness (5 min):**   - Define belonging and uniqueness (2 min) - Describe Shore et al. 2x2 framework and how these improve inclusivity (2 min) - Discuss how sharing personal stories (storytelling) can enhance both belonging and uniqueness (1 min) |
| **Individual and Small Group Activity (4 min):**   - Query the audience to reflect on a story or experience that was meaningful to them (2 min) - Pair-share with a neighbor or small group (2 min) |
| **Didactic - Introduction to Communication Model (10 min):**   - Describe the history of the SPIKES model of communication and the components of the framework (4 min) - Describe how we can adapt and apply the SPIKES model of communication to a personal or professional experience (6 min**)** |
| **Presentation of Personal Experiences (8 min):**   - Each presenter has the chance to choose a personal experience and apply the SPIKES model of communication and how it relates to creating a culture of belonging (2-4 min/presenter)   **Small Group Activity (1-2 min):**   - Reflect back on initial prompt of “what does belonging mean to you?” How has this changed throughout the workshop and listening to other’s stories? Pair-share with a neighbor. |
| **Individual and Small Group Activity (15 min):**   - Participants will work on their own to use the SPIKES model of communication to apply to their own personal story (3 min) - Participants will share their stories through pair-share with a neighbor or small group (7 min) - Ask a few participants to share their stories with the large group (5 min) |
| **Individual and Small Group Activity (5 min):**   - Ask audience reflection questions and to pair-share with a neighbor or small group - Ask a few participants to share their reflections with the large group |
| **Conclusions (6 min):**   - Workshop take home points (1 min) - Call to Action (2 min) - Evaluation (3 min) |
