# Supplementary material for: SPIKING A Sense of Belonging: Utilizing a Communication Model to Unlock Your Story With Authenticity
Source: MedEdPORTAL. 2025 Dec 30;21:11567. doi: 10.15766/mep_2374-8265.11567 (PMC12748279; doi:10.15766/mep_2374-8265.11567)
Supplement: Supplementary file 1 — Workshop Presentation.pptxFacilitator Guide.docxHandout.docxPresentation Script.docxEvaluation Form.docx [file mep_2374-8265.11567-s001.zip › C. Handout.docx]

Adapted SPIKES Model of Communication for Storytelling (Appendix C)
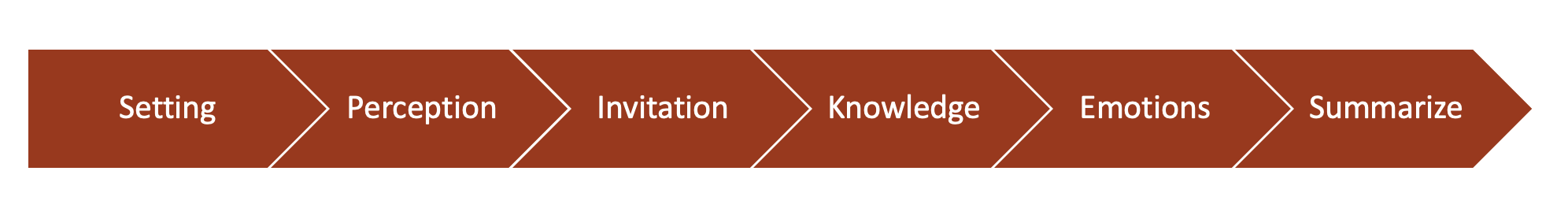


How to Implement the SPIKES Model of Communication:

- - **Setting:** Describe the setting of your story. This can be the setting of where your story starts or ends. Set the stage for your audience, teammates, or working group with whom you are sharing your story. This can be a period of time, a place, or a direction (past, present, or future).
  - **Perception:** Perception can mean different things. It may reflect what your audience already knows about you, their understanding of your story, or your own perception of the events you describe. Utilize many of these angles to engage your audience and gain feedback about your story you are sharing.
  - **Invitation:** This relates to inviting different emotions that arise while you are telling your story, as well as inviting the audience to relate or ask questions about what you share. Welcoming others in, opens your world to common interests, shared perspectives, and future connections.
  - **Knowledge:** Knowledge refers to the knowledge you have learned through experiences shared within your story, and how those lessons shape your outlook on the future. Consider what you hope others will learn about you and their surroundings by hearing your story.
  - **Emotions:** Sharing stories can bring up a range of emotions, both positive and negative. Allow this part of the communication model to be fluid as you and your audience respond to what is being shared.
  - **Summarize:** Conclude by reflecting on what you want your audience to take away from your story. Summarize what you have learned by sharing your experience and what you hope to gain from revealing a personal or professional part of your journey.
