# Supplementary material for: SPIKING A Sense of Belonging: Utilizing a Communication Model to Unlock Your Story With Authenticity
Source: MedEdPORTAL. 2025 Dec 30;21:11567. doi: 10.15766/mep_2374-8265.11567 (PMC12748279; doi:10.15766/mep_2374-8265.11567)
Supplement: Supplementary file 1 — Workshop Presentation.pptxFacilitator Guide.docxHandout.docxPresentation Script.docxEvaluation Form.docx [file mep_2374-8265.11567-s001.zip › D. Presentation Script.docx]

**SPIKING A Sense of Belonging: Utilizing a Communication Model to Unlock Your Story with Authenticity**

*PowerPoint Presentation Script & References*

**Slide 1:** Title Slide & Introduction

- Introduction of title and authors

**Slide 2:** Disclosures

- Present financial or academic disclosures

**Slide 3:** Learning Objectives

- ***Differentiate*** belonging and uniqueness.
- ***Illustrate*** how sharing personal stories can enhance a sense of belonging in the workplace.
- ***Describe*** how the SPIKES model of communication can be adapted to sharing personal stories.
- ***Apply*** the SPIKES model of communication to share your own personal story to foster a sense of authenticity and belonging in the workplace.

**Slide 4:** Group Activity: “Tell me about yourself”

- Prompt the workshop participants to write down a statement telling the audience about themselves and reflecting on what belonging means to them.
- This can be a full introduction with a few sentences to just a few words that resonate with them. Some examples can include, “I am a professor of anesthesiology,” “I am a graduate student studying neuroscience,” “I am from California, and I am a radiology resident.” The presenters can share their own descriptions as an example.
- After a moment of individual reflection, ask participants to pair-share with their neighbor or in small groups.
- Depending on time, can ask a few volunteers to share with the large group their reflections on what belonging meant to them.
- The aim of this is to get participants thinking about how their introductions to someone new in the workplace allows them to tell their story. We will revisit this concept later in the workshop.

**Slide 5:** Definitions

- Define what *belonging* and *uniqueness* are – you can use the definitions outlined on the slide vs. dictionary definition.

**Slide 6:** Explanation of Inclusion Framework

- **References:**
  - Shore LM, Randel AE, Chung BG, Dean MA, Holcombe Ehrhart K, Singh G. Inclusion and Diversity in Work Groups: A Review and Model for Future Research. *Journal of Management*. 2011;37(4):1262-1289. doi:10.1177/0149206310385943.
  - Ely RJ, Thomas DA. Cultural Diversity at Work: The Effects of Diversity Perspectives on Work Group Processes and Outcomes. *Administrative Science Quarterly*. 2001;46(2):229-273. doi:10.2307/2667087
  - Friedman R, Kane M, Cornfield DB. Social Support and Career Optimism: Examining the Effectiveness of Network Groups Among Black Managers. *Human Relations*. 1998;51(9):1155-1177. doi:10.1177/001872679805100903
- The goal of this slide is to explain the inclusion framework of Shore et al.
- Shore et al. describes a framework of inclusion that proposes that the intersection of uniqueness and belonging creates a feeling of inclusion. Individuals feeling a sense of high belongingness and high value in uniqueness are treated as an ‘insider’ and benefit from being a unique member of a group. On the other end of the framework, those who feel low belongingness and low value in uniqueness are excluded. They are often treated as an ‘outsider,’ one who is not valued for their unique qualities. Those who have a sense of high belongingness, but low value in uniqueness, assimilate and conform to organizational or dominant culture norms, which also continue to downplay uniqueness. Those with low belongingness and high value of uniqueness are often valued for their unique qualities but are not treated as an ‘insider’. Ely and Thomas’s qualitative study illustrated that work groups who adopted this perspective acknowledged the unique qualities of individuals, but minority members were not considered to be a part of the larger organization and therefore, were subject to isolation and stereotypes.
- There is support in the diversity literature for the advantages of experiencing belongingness and uniqueness simultaneously. For example, minority members (who are unique) with developed networks (and thus a sense of belongingness) report a high level of career optimism (Friedman, Kane, & Cornfield, 1998). At the group level, diverse work groups that adopt an integration-and-learning perspective incorporate both uniqueness (through viewing diversity as a resource) and belongingness (through members feeling valued and respected; Ely & Thomas, 2001). Work groups that adopt an integration-and-learning perspective demonstrate high-quality analyses, are able to facilitate effective cross-organizational collaboration, and allow individuals within the group to enhance their skills (Ely & Thomas, 2001).
- Studies show that fostering belonging and uniqueness together benefits diverse team members through increased career optimism, group collaboration, and the augmentation of individual skills.

**Slide 7:** Explanation of how storytelling fits within the inclusion framework

- **References:**
  - Haggins AN. To Be Seen, Heard, and Valued: Strategies to Promote a Sense of Belonging for Women and Underrepresented in Medicine Physicians. *Academic Medicine*. 2020;95(10):1507-1510. doi:10.1097/ACM.0000000000003553
  - Bickel J, Rosenthal SL. Difficult Issues in Mentoring: Recommendations on Making the “Undiscussable” Discussable: *Academic Medicine*. 2011;86(10):1229-1234. doi:10.1097/ACM.0b013e31822c0df7
- Storytelling or sharing one’s personal stories, historically has served as a tool for self-reflection, healing and connecting with others.
- Through storytelling, we invite others to empathize with the challenges, triumphs, and shared personal experiences one may encounter.
- There is a critical need to create spaces for collective dialogue among colleagues, medical residents, and students about the intersection of their personal (e.g. race, ethnicity, and gender), and professional identities.
- While storytelling holds tremendous potential to foster connection and belonging, effectively sharing one’s story—particularly in professional (medicine), cross-cultural spaces—requires more than willingness alone.
- Many individuals lack the frameworks, language, or confidence to articulate their lived experiences in ways that feel both authentic and professionally appropriate.
- Through storytelling, the intersection of belonging and uniqueness foster inclusivity.

**Slide 8:** Group Activity: “Reflect on a story or experience that was meaningful to you?”

- Query the audience another question, “Reflect on a story or experience that was meaningful to you and why?” Engage the audience to write down their answers as they are reflecting. In small groups or a peer-to-peer breakout session, instruct the workshop participants to share what they wrote with a neighbor. Allow enough time for both individuals and small group members to share their story/experience in a small group setting. Ask the audience for volunteers to share what their story was. If no participants start, offer to present your own thoughts about a story or experience that was meaningful to you.
- Encourage the audience to share their story in whatever capacity they feel comfortable.

**Slide 9:** Introduction to the SPIKES Model of Communication

- **References:**
  - Baile WF, Buckman R, Lenzi R, Glober G, Beale EA, Kudelka AP. SPIKES—A Six-Step Protocol for Delivering Bad News: Application to the Patient with Cancer. *The Oncologist*. 2000;5(4):302-311. doi:10.1634/theoncologist.5-4-302
  - McFarlane J, Riggins J, Smith TJ. SPIKES: A Six-Step Protocol for Delivering Bad News About the Cost of Medical Care. *JCO*. 2008;26(25):4200-4204. doi:10.1200/JCO.2007.15.6208
  - Kaplan M. SPIKES: A Framework for Breaking Bad News to Patients with Cancer. *Clinical Journal of Oncology Nursing*. 2010;14(4):514-516. doi:10.1188/10.CJON.514-516
  - Mahendiran M, Yeung H, Rossi S, Khosravani H, Perri GA. Evaluating the Effectiveness of the SPIKES Model to Break Bad News – A Systematic Review. *Am J Hosp Palliat Care*. 2023;40(11):1231-1260. doi:10.1177/10499091221146296
- Many individuals lack frameworks, language, or confidence to articulate their lived experiences in ways that feel both authentic and professionally appropriate. Just as we train clinicians in communication and procedural skills, we must also intentionally cultivate the skills, provide the tools, and offer structured practice environments that empower individuals to share their narratives with clarity, purpose, and impact.
- We introduce the SPIKES model as a communication method that can be adapted to storytelling in the workplace to enhance uniqueness and belongingness.
- This is a six-part framework that sets out a simple process for sharing difficult to hear news with patients and their families.
- Dr. Robert Buckman, Dr. Michael Levy, Dr. Walter Baile developed the model of communication, and it was first presented in 1998 at the American Society of Clinical Oncology.
- Studies have shown that this skill can be taught.
- This form of communication can be used globally in a variety of clinical situations, as well as to improve communication and expectations in the workplace.

**Slide 10 – 12:** Breaking down the SPIKES Model of Communication

- Explain each letter in the original SPIKES Model of Communication (Slide 10-11)
- Explanations for the adapted SPIKES Model of Communication for Storytelling specifically (Slide 12)

**Slide 13:** Applying the SPIKES model of communication to a personal or professional experience

- Prompt the audience with these questions as presenters transition to applying the SPIKES model of communication to their own personal or professional experiences.
  - How can we apply the six-part model of communication to a personal or professional experience in the workplace?
  - Are we able to extract the principles of this method of communication to enhance a sense of belonging?

**Slide 14 – 16 +:** Presenters will use these slides to tell their personal story using the SPIKES model of communication (see below for an example)

- Now engage each presenter to present their unique experience using the SPIKES model of communication:
- Step-by-step guide for implementing the SPIKES model of communication to your personal or professional experience.
  - **Setting:** Describe the setting of your story. This can be the setting of where your story starts or ends. Set the stage for your audience, teammates, or working group with whom you are sharing your story. This can be a period of time, a place, or a direction (past, present, or future). **– Ex. An undergraduate student who began her career as a ballet dancer, ultimately getting injured and finding her way to an academic medical career. As an injured dancer, she became the patient in the patient-physician relationship and later aspired to be the one taking care of patients.**
  - **Perception:** Perception can mean different things. It may reflect what your audience already knows about you, their understanding of your story, or your own perception of the events you describe. Utilize many of these angles to engage your audience and gain feedback about your story you are sharing. **– Ex. The perception of how the endurance, resilience, dedication, and tenacity developed through ballet training, shaping her personality to succeed in medical school and residency training.**
  - **Invitation:** This relates to inviting different emotions that arise while you are telling your story, as well as inviting the audience to relate or ask questions about what you share. Welcoming others in, opens your world to common interests, shared perspectives, and future connections. **– Ex. Inviting the audience to relate to similar hardships of giving up a part of one’s identity to form a new one and exploring how this shapes us moving forward.**
  - **Knowledge:** Knowledge refers to the knowledge you have learned through experiences shared within your story, and how those lessons shape your outlook on the future. Consider what you hope others will learn about you and their surroundings by hearing your story. **– Ex. Drawing on her experiences as a ballerina, she carried forward her work ethic, discipline, and dedication to build an academic career. She used those lessons learned to connect and advocate for patients and to continue expressing creativity and artistry in her medical career.**
  - **Emotions:** Sharing stories can bring up a range of emotions, both positive and negative. Allow this part of the communication model to be fluid as you and your audience respond to what is being shared. **– Ex. Feelings of loss and grief after her identify changed were accompanied by excitement, passion for medicine and the fulfillment in helping others while sharing her unique background with others.**
  - **Summarize:** Conclude by reflecting on what you want your audience to take away from your story. Summarize what you have learned by sharing your experience and what you hope to gain from revealing a personal or professional part of your journey**. – Ex. You never know what type of impact your personal story might have on someone else. Sharing these vulnerabilities may help a patient or colleague in ways you never imagined.**
- **Include personal photos, visuals, quotes, videos to illustrate how you are utilizing the SPIKES model of communication and applying it to your own experience (be creative).**
- As presenters create their SPIKES model of communication story, it helps to include photographs or other graphics to individualize the story

**Slide 17:** Group Activity

- Prompt the audience by asking them to reflect back on “what does belonging means to you” from the beginning of the workshop.
- After hearing the presenter’s stories, does the meaning of belonging change?
- Ask participants to pair-share with their neighbor or in small groups. You can prompt groups to share if they feel comfortable.

**Slide 18:**  Your Turn! Using the SPIKES model of communication for Your Story:

- Now in small groups, we ask workshop participants to mirror the presenters and use the SPIKES model of communication application to their own personal or professional experience.
- Encourage participants to use the SPIKES model of communication handout (Appendix C) while they are thinking about their story.
- You can also have participants write out the SPIKES model of communication with their personal experiences to encourage volunteers to share with the larger group.
- Allow some time for participants to share with their peers in small groups before opening it up to the larger group.

**Slide 19:** Group Activity: Reflections

- Ask audience reflection questions and to pair-share with a neighbor or small group
- Ask a few participants to share their reflections with the large group
  - How did that make you feel?
  - What was it like applying the SPIKES model of communication to your story?
  - Have you discussed your story with others you work with? Why or why not?
  - Do you feel comfortable sharing with others after this exercise?

**Slide 20:** Re-visiting our Learning Objectives

- Return to the learning objectives to summarize what the audience learned from participating in this workshop.

**Slide 21:** Key Take Home Points:

- Balancing belonging with uniqueness, particularly in diverse group settings like medicine, remains a **challenge** that can threaten inclusion.
- High belonging and high uniqueness illustrate **inclusion** in an organization.
- **Storytelling** can be a powerful way to cultivate inclusion. However, many individuals lack frameworks, language, or confidence to share their stories in ways that feel both authentic and professionally appropriate in the workplace.
- Recognizing that many individuals struggle with how to structure and share emotionally complex or deeply personal narratives, we saw parallels between the challenges of disclosing difficult news to patients and the vulnerability involved in sharing one's own story.
- We adapted the **SPIKES** model of communication as a supportive framework for how people could share their personal and professional stories to foster a sense of belonging and cultivate authenticity among colleagues.

**Slide 22:** Call to ACTION: You BELONG!

- Our workshop addresses a persistent gap in medical education, where there is a lack of inclusive communication strategies taught to share personal stories. Research shows that underrepresented individuals in academic medicine, especially women and racial and ethnic minorities, continue to experience disrespect and exclusion. The National Science Foundation continues to draw attention to the alarming rate of harassment, incivility, and disrespect among underrepresented individuals in academic medicine, and that other racial and ethnic groups are at risk of being targeted.
- We challenge the audience to think about what actions they are going to take from participating in this workshop. This is a call-to-action in which we urge workshop participants to continue practicing vulnerability in sharing stories. Spread the word of belonging and be the person that creates this change.

**Slide 23:** References

**Slide 24:** Acknowledgements
