# Supplementary material for: SPIKING A Sense of Belonging: Utilizing a Communication Model to Unlock Your Story With Authenticity
Source: MedEdPORTAL. 2025 Dec 30;21:11567. doi: 10.15766/mep_2374-8265.11567 (PMC12748279; doi:10.15766/mep_2374-8265.11567)
Supplement: Supplementary file 1 — Workshop Presentation.pptxFacilitator Guide.docxHandout.docxPresentation Script.docxEvaluation Form.docx [file mep_2374-8265.11567-s001.zip › E. Evaluation Form.docx]

**EVALUATION FORM**

**SPIKING A Sense of Belonging: Utilizing a Communication Model to Unlock Your Story with Authenticity**

|  | Strongly  Disagree | Disagree | Neutral | Agree | Strongly Agree |
| --- | --- | --- | --- | --- | --- |
| Workshop met objective #1:  ***Differentiate*** belonging and uniqueness. | 1 | 2 | 3 | 4 | 5 |
| Workshop met objective #2:  ***Illustrate*** how sharing personal stories can enhance a  sense of belonging in the workplace. | 1 | 2 | 3 | 4 | 5 |
| Workshop met objective #3:  ***Describe*** how the SPIKES model of communication can  be adapted to sharing personal stories. | 1 | 2 | 3 | 4 | 5 |
| Workshop met objective #4:  ***Apply*** the SPIKES model of communication to share your own personal story to foster a sense of authenticity and belonging in the workplace. | 1 | 2 | 3 | 4 | 5 |
| Workshop was a valuable use of my time. | 1 | 2 | 3 | 4 | 5 |
| I will apply information learned today. | 1 | 2 | 3 | 4 | 5 |

What are two things you will do differently as a result of this workshop?

1.

2.

What do you see as potential barriers to applying what you have learned?

What did you like best about the workshop?

What can we improve about the workshop?

*The following questions are to learn more about who participants in the workshop are. Responses will be aggregated and not linked to an individual. Please circle the response that best describes you.*

| Role | Race & Ethnicity (can select multiple) | Gender |
| --- | --- | --- |
| Trainee (student, resident, fellow)  Faculty  Educational or Admin Staff  Clinical Staff  Prefer Not to Answer  Prefer to Self-Describe (free text): | American Indian or Alaska Native  Asian  Black or African American  Hispanic or Latino/a/x  Native Hawaiian or Pacific Islander  White  Prefer Not to Answer  Prefer to Self-Describe (free text): | Cisgender Man  Cisgender Woman  Gender Non-Conforming/Non-Binary  Transgender Man  Transgender Woman  Prefer Not to Answer  Prefer to Self-Describe (free text): |
